# Impacts of COVID-19 on clinical indicators and mortality in patients with chronic conditions in Catalonia, Spain: A retrospective population-based cohort study

**DOI:** 10.7189/jogh.14.05020

**Published:** 2024-06-21

**Authors:** Manuel Moreno-Vásquez, Josep Vidal-Alaball, Marc Saez, Maria A Barceló, PERIS Project collaborative team

**Affiliations:** 1 Research Group on Statistics, Econometrics and Health (GRECS), University of Girona, Girona, Spain; 2 Unitat de Suport a la Recerca de la Catalunya Central, Fundació Institut Universitari per a la Recerca a l’Atenció Primària de Salut Jordi Gol i Gurina, Manresa, Barcelona, Spain; 3 Centro de Investigación Biomédica en Red de Epidemiología y Salud Pública, Instituto de Salud Carlos III, Madrid, Spain; 4 Health Promotion in Rural Areas Research Group, Gerencia d’Atenció Primària i a la Comunitat de la Catalunya Central, Institut Català de la Salut, Manresa, Barcelona, Spain; 5 Faculty of Medicine, University of Vic – Central University of Catalonia, Vic, Barcelona, Spain

## Abstract

**Background:**

The reallocation of health care services during the coronavirus disease 2019 (COVID-19) pandemic disrupted the continuity of primary care. This study examines the repercussions of the COVID-19 pandemic on clinical indicators within the Catalan population, emphasising individuals with chronic conditions. It provides insights into mortality and transfer rates considering intersectional perspectives.

**Methods:**

We designed a retrospective, observational population-based cohort study based on routinely collected data from January 2015 to June 2021 for all individuals available in the Information System for Research in Primary Care (*Sistema d'Informació per al Desenvolupament de la Investigació en Atenció Primària* (*SIDIAP*)), the largest public primary care database in Catalonia, Spain. We included 6 301 095 individuals, constituting 81.6% of Catalonia's population in 2020. To perform a repeated measurements analysis of the indicators, we focussed on individuals who had one or more indicators in both the pre-pandemic (January 2015 to March 2020) and pandemic periods (March 2020 to June 2021), and those diagnosed with type 2 diabetes mellitus (T2D), high blood pressure, and heart failure. We selected key clinical indicators for analysis, including systolic and diastolic blood pressure, body mass index (BMI), cholesterol (total, high, and low-density lipoprotein), triglycerides, glycosylated haemoglobin, the Barthel index, and cardiovascular risk (*Registre Gironí del cor* (*REGICOR*) index).

**Results:**

Mortality and transfer rates increased during the pandemic, contributing to a decline in the active population in the public health system. We also observed a reduction in pandemic period prevalence of patients with chronic conditions: −26.7% for heart failure, −15.1% for high blood pressure, and −14.6% for T2D. In both pre-pandemic and pandemic periods, 1 632 013 subjects had at least one clinical indicator record. Clinical indicators worsened in patients diagnosed with chronic conditions during the pandemic. Most indicators worsened, with differences between men and women (+9.4% vs +3.7% for the *REGICOR* index and −14.1% vs −16.6% for the Barthel index in men and in women, respectively), and to a similar extent (or greater in some cases) in individuals without these conditions.

**Conclusions:**

We used longitudinal data to assess the repercussions of the COVID-19 pandemic on population health while considering a wide range of clinical indicators and socioeconomic determinants. Our analysis shows a deterioration in clinical indicators during the pandemic, particularly in cardiometabolic factors, underscoring the importance of continuous primary care for individuals with chronic conditions.

Since the beginning of 2020, the coronavirus disease 2019 (COVID-19) pandemic has affected over 762 million people worldwide, causing almost 7 million deaths [1]. People with cardiovascular and metabolic conditions were especially vulnerable due to their higher risk of severe COVID-19 infection and mortality [2]. Moreover, the reallocation of health care resources from chronic to COVID-19 care disrupted the continuity of treatment for these patients, leading to reductions in face-to-face consultations (−42%), diagnoses (−31%), and therapeutics (−30%) [3]. This disruption resulted in higher morbidity among chronic patients, including an increase in hyperglycaemic and hypertensive emergencies, acute decompensated heart failure cases [4], and a predicted rise in cardiovascular events such as myocardial infarction and stroke [5].

Furthermore, socioeconomic factors and gender inequalities may have exacerbated the pandemic’s negative consequences for chronic patients. Populations with lower income or socioeconomic status have experienced higher morbidity and mortality rates due to COVID-19 and significant delays in health care accessibility compared to those with higher income and status [6,7]. Additionally, while men experienced more severe morbimortality from COVID-19, the pandemic has amplified existing gender disparities, with women being disproportionately affected in terms of health care provision, employment, or mental health [8,9].

Thus far, some studies have examined the COVID-19 pandemic’s socioeconomic impact using ecological designs [10], while others have analysed its effect on chronic care using large primary care data repositories [11–13]. However, these evaluations were either limited to short periods [11] or specific clinical indicators [12,13]. To date, there has been no longitudinal study of the pandemic’s influence on chronic diseases, considering various clinical indicators, a substantial timeframe spanning both pre-pandemic and pandemic periods, and potential socioeconomic or gender-based disparities.

To address this need, we conducted a retrospective population-based cohort study encompassing both the pre-pandemic and pandemic periods. Our overall goal was to assess the impact of the COVID-19 pandemic on population health, considering intersectional and sex perspectives. For this purpose, we used routinely collected data from Catalonia’s largest primary care database, the Information System for Research in Primary Care (*Sistema d'Informació per al Desenvolupament de la Investigació en Atenció Primària (SIDIAP*)) [14]. Our primary objective was to compare clinical indicators before and during the pandemic, categorised by most prevalent chronic diseases and by sex. Our secondary objective was to describe socioeconomic factors that influenced the mortality and transfer rates during the pandemic period.

## METHODS

### Study design, data source, and population

We conducted a retrospective observational population-based cohort study using data from all patients from *SIDIAP*, for the period from 1 January 2015 and 30 June 2021. *SIDIAP* is the largest primary care database in Catalonia, Spain, and comprises extensive information from 328 primary care centres managed by the *Institut Català de la Salut*, covering records on demographics, visits, diagnoses, laboratory tests, and drug prescriptions for approximately 6.3 million people [14], which represents 81.6% of the population of Catalonia. The data set is organised into data domains, each with a unique pseudo-anonymised code assigned to individuals for linking purposes [14].

The pre-pandemic period in our analysis ends on 13th March 2020, the date when Spain’s government declared the first state of alarm, leading to a major shift in primary care consultations. The nationwide lockdown is treated as the onset of the pandemic period which is close to the first wave of COVID-19 cases. The pandemic period included three waves: from March to the end of May 2020; from October to mid-November 2020; and from the end of November 2020 to the end of February 2021.

The definition of both periods (pre-pandemic and pandemic) determined the main inclusion criteria for the repeated measurements analysis, whereby at least one clinical indicator record had to have been available for subjects in the pre-pandemic and pandemic periods for them to be included in our study.

The Ethics Committees of *Fundació Institut Universitari per a la Investigació en Atenció Primària de Salut Jordi Gol i Gurina* approved our study (22/085-PCV). We did not require written informed consent, as patient data extracted from the database were irreversibly pseudonymised. We reported our study per the REporting of studies Conducted using Observational Routinely collected Data (RECORD) checklist [15], which is an extension of the STrengthening the Reporting of OBservational studies in Epidemiology (STROBE) guidelines [16] (Table S1 in the **Online Supplementary Document**).

### Variables

We define, as a status variable, the situation of the subject during the follow-up period (active, deceased, or transferred out of Catalonia’s health system) (Table S2 in the **Online Supplementary Document**).

Sociodemographic variables at the patient level were sex, age (categorised into subgroups), region, urbanicity, the *Mortalidad en áreas pequeñas Españolas y Desigualdades Socioeconómicas y Ambientales* (*MEDEA*) deprivation index, and prescription cost-sharing contribution variables (used as proxies for annual income categorisation) (Table S2 in the **Online Supplementary Document**).

Regarding clinical variables, we selected the most prevalent chronic diseases monitored by primary health care services in *SIDIAP* using the 10th revision of the International Statistical Classification of Diseases and Related Health Problems codes (ICD-10) [17]. These were type 2 diabetes mellitus (T2D), high blood pressure (HBP) or hypertension, and heart failure (HF) (Table S2 in the **Online Supplementary Document**).

For the analysis of the repeated measurements, we retrieved key clinical indicators related to these chronic diseases, including systolic and diastolic blood pressure, body mass index (BMI), total cholesterol, high-density lipoprotein cholesterol (HDL-C), low-density lipoprotein cholesterol (LDL-C), triglycerides, fasting blood glucose/glycosylated haemoglobin (HbA1c), geriatric assessment (the Barthel index), cardiovascular risk (*Registre Gironí del cor* (*REGICOR*) index), and smoking status. In cases where more than one indicator value before and during the pandemic periods was available, we used the most recent within each of them (Table S2 in the **Online Supplementary Document**).

We obtained data on the Basic Health Area (*Areas Básicas de Salud* (*ABS*)) net income and Gini index from other sources (Table S3 in the **Online Supplementary Document**).

### Statistical analysis

As this was a population-based study, a formal calculation of the sample size was not applicable. We included subjects in the repeated measurements analysis according to their conformity to the inclusion criteria, making each of them their control and thus reducing bias. We reported categorical variables as absolute and relative frequencies, and quantitative variables as means and standard deviations. In the descriptive analysis, we grouped sociodemographic data and prevalent diagnoses for the entire population in the pre-pandemic and pandemic periods. Additionally, we calculated the monthly number of deaths and transfers, as well as the corresponding rates (expressed as percentages) from January 2018 to June 2021.

We also grouped clinical indicators in the two study subperiods in the repeated measurements analysis and stratified them by sex and main diagnosis (T2D, HBP, and HF) as follows: diagnosis (yes and no) and time of diagnosis (before and during the pandemic). We compared paired data from clinical indicators obtained in the pre-pandemic and pandemic periods using the Wilcoxon signed rank test. We used a non-parametric test because the normality assumption of the quantitative variables partitioned by periods (pre-pandemic and pandemic) was rejected in all cases (per the Shapiro-Wilk normality test). We did not use any imputation methods for missing data. The significance threshold was set at a two-sided α<0.05. We performed all analyses in R, version 4.2.2 (R Core Team, Vienna, Austria).

### Data access

The study investigators had access to *SIDIAP* to extract irreversibly pseudonymised data. In compliance with the study protocol and Spanish legislation (Law 3/2018, of 5 December, on Personal Data Protection and Guarantee of Digital Rights), data transfer to third parties was restricted and data are not publicly available.

### Role of the funding source

This project was carried out with the support of the Department of Health of the Government of Catalonia and the *Agencia de Gestión de Ayudas Universitarias y de Investigación*, Government of Catalonia. The funding sources did not participate in the design or conduct of the study; the collection, management, analysis, or interpretation of the data; or the preparation, review, and approval of the manuscript.

## RESULTS

### Sociodemographic characteristics of cohort and most prevalent diagnoses

We included 6 301 095 individuals in the descriptive analysis, accounting for 81.6% of Catalonia’s population in 2020 [18]. Among the entire cohort, 1 632 013 individuals (25.9%) had clinical indicator records before and during the pandemic and were selected for the repeated measurements analysis (Figure S1 in the **Online Supplementary Document**).

**Figure 1:**
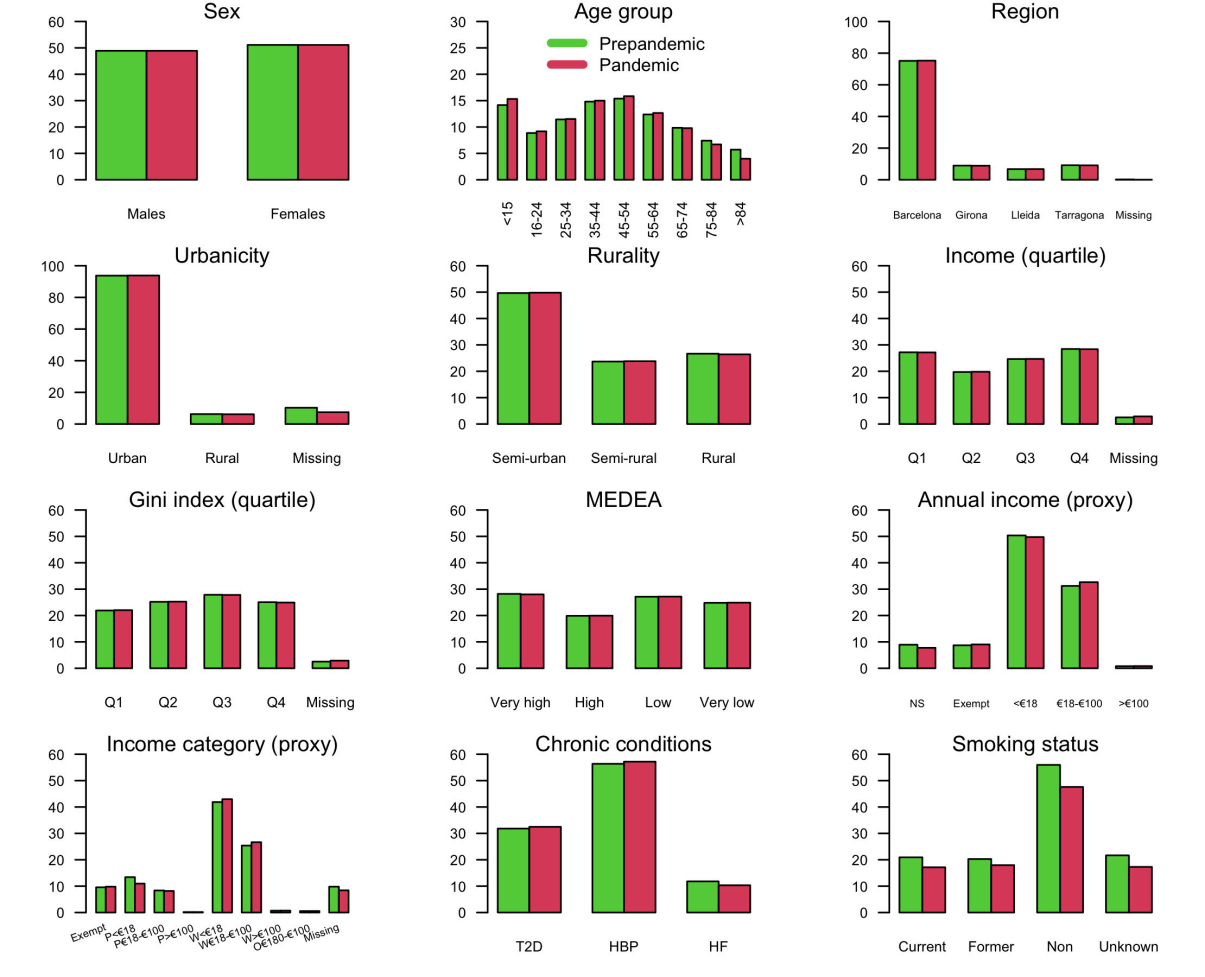
Percentages of demographics and prevalent chronic diseases for the entire *SIDIAP* population. €<18 – EUR<18 000,>€100 – EUR>100 000, 18€–€100 – EUR 18 000 to EUR 100 000, *MEDEA* – *Mortalidad en áreas pequeñas Españolas y Desigualdades Socioeconómicas y Ambientales*, O – others (excluded), P – pensioners, Q – quartile, W – workers.

Overall, 51.1% of the included individuals were women and 22.6% were aged 65 or older (**Figure 1** and Table S4 in the **Online Supplementary Document**). Subjects over 65 years of age and, especially those over 74 years of age among them, experienced the greatest population decrease during the pandemic (from 22.9% to 20.5%). Most individuals lived in Barcelona (75.0%) and urban areas (85.1%), and 52.0% of the urban population had a low (27.1%) or very low (24.9%) socioeconomic level according to the *MEDEA* deprivation index. More than half fell into lower socioeconomic status categories (3rd and 4th quartiles) according to the income and Gini indexes, which is consistent with approximately 50% of the population having a low income (EUR<18 000). During the pandemic, the percentage of individuals with low income decreased slightly from 50.4% to 49.7%. Pensioners accounted for nearly 20% of the population. This group experienced a notable drop in their proportion within the population during the pandemic (from 20.0% to 17.8%), particularly among individuals with low incomes (from 12.2% to 10.1%). Overall, the period prevalence of chronic conditions decreased during the pandemic period by 14.6% for T2D (from 11.8% to 10.1%), 15.1% for HBP (from 20.8% to 17.7%), and 26.7% for HF (from 4.4% to 3.2%). Additionally, the percentage of current smokers decreased slightly from 15.9% to 15.3%.

### Evolution of mortality and transfer rates

**Figure 2:**
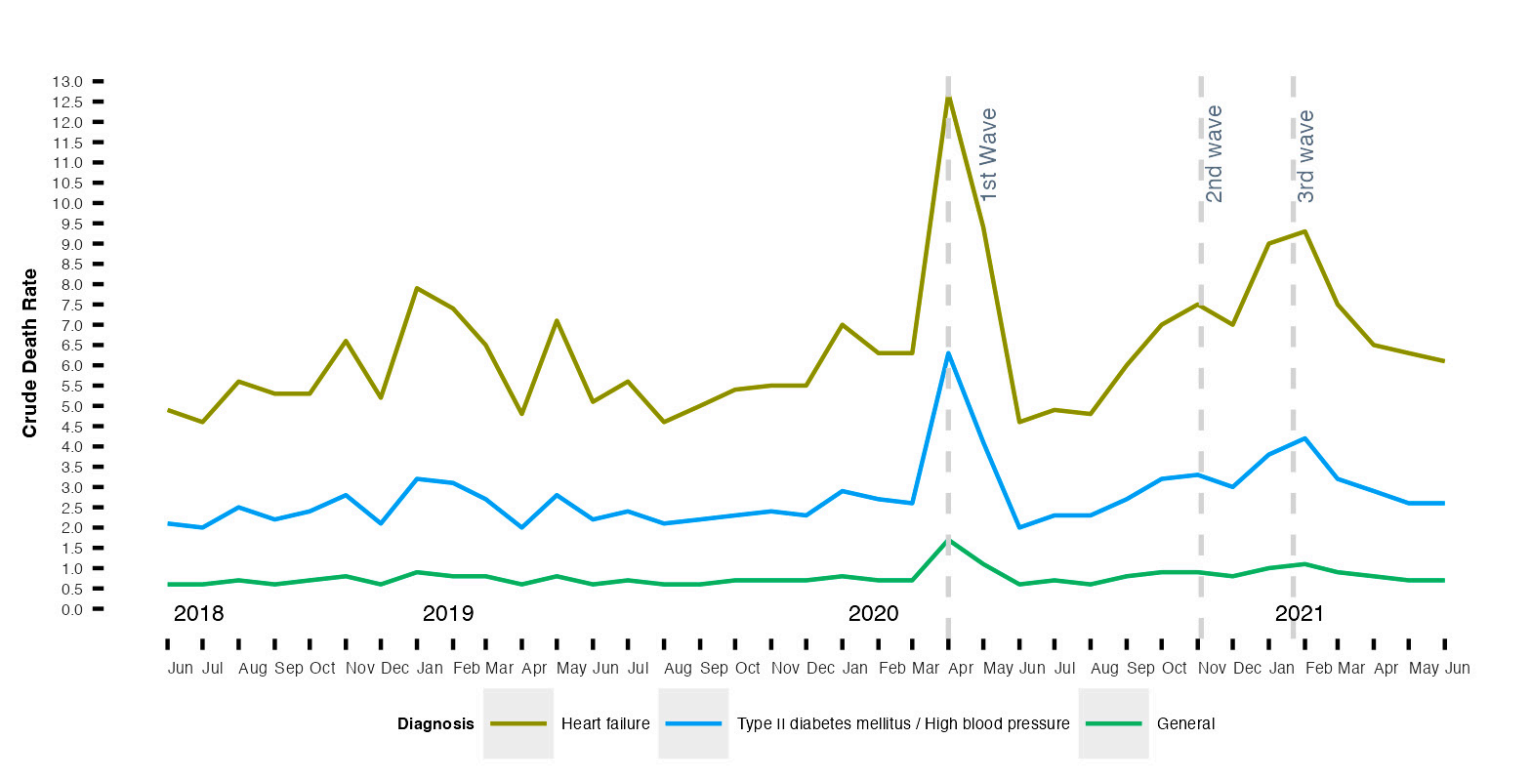
Crude monthly mortality rate per 1000 inhabitants among subjects diagnosed with HBP, T2D, and HF, as well as in the general population. The rates of T2D and HBP are approximately equal, which is why both rates are merged in the figure.

On 13 March 2020, 5 672 955 individuals were active in the public health system. Of this population, 1.3% died in the pandemic period and 1.9% were transferred out of the Catalonian health system. During the pandemic, on 30 June 2021, there was 5 593 333 active population, presenting a reduction of 79 622 (1.4%) active cohort subjects. Mean mortality rates increased from 0.07% to 0.17% from the pre-pandemic to the first wave period and decreased subsequently in the second (0.09%) and third waves (0.11%). Individuals over 85 years of age experienced the largest increase in mortality rates, particularly in the first wave (from 1% to 2.5%). Stratification by income showed that pensioners with low income were the most affected, with mean mortality rates rising from 0.4% to 1.1% in the first wave. Patients in the 4th quartile of the *MEDEA* deprivation index were the most affected in the third wave, with an average mortality rate of approximately 0.15%, compared to approximately 0.10% for individuals in other quartiles (Figure S2 in the **Online Supplementary Document**). Although no information regarding the cause of mortality of the subjects in our study is available, we managed to determine the monthly crude mortality rate for people diagnosed with each of the studied chronic diseases, as well as for the general population (**Figure 2**). During the first wave of the pandemic, the rate roughly doubled in the population diagnosed with HF, T2D, and HBP. During the second and third waves of the pandemic, we observed the highest increase in crude death rate for HF patients. This is in contrast with the disaggregated general population, where we only observed a sharp increase in the death rate in the first wave. When comparing the average annual mortality rate pre-pandemic (T2D = 2.3%, HBP = 2.4%, HF = 4.8%) and during the pandemic (T2D = 3.7%, HBP = 3.7%, HF = 7.8%), we observed a significant increase, especially in HF patients (Table S5 in the **Online Supplementary Document**).

Additionally, the average transfer rate increased by 0.04%, from a mean monthly transfer rate of 0.08% before the pandemic to 0.12% during the pandemic, particularly in the 25–34 and 35–44 age groups (from 0.08% to 0.12%) (Figure S3 in the **Online Supplementary Document**).

### Repeated measurements analysis of clinical indicators before and during the pandemic according to sex (primary objective)

All indicators besides HDL-C and LDL-C worsened during the pandemic, with overall moderate variations. However, these were substantial for the *REGICOR* index, with a 7.2% increase, and the Barthel index, with a 15.7% decrease (**Table 1**).

**Table 1:** Comparison of clinical indicators before and during the pandemic overall and stratified by sex

Clinical indicators (n)	Pre-pandemic, x̄ (SD)	Pandemic, x̄ (SD)	Difference (%)*
Cholesterol			
*Total (1 508 616)*	195.7 (41.7)	197.1 (41.7)	1.4 (0.7)
*Men* (628 425)	187.7 (41.6)	187.6 (41.6)	−0.1 (0.0)
*Women (880 191)*	201.4 (40.8)	203.9 (40.8)	2.5 (1.2)
HDL-C			
*Total (1 048 253)*	55.8 (14.7)	56.9 (14.7)	1.1 (2.0)
*Men (451 461)*	49.9 (12.5)	50.8 (12.5)	1.0 (1.9)
*Women (596 792)*	60.3 (14.6)	61.6 (14.6)	1.2 (2.0)
LDL-C			
*Total (1 047 481)*	119.2 (36)	118.3 (36)	−1.0 (−0.8)
*Men (450 923)*	113.3 (36.0)	111.4 (36)	−1.9 (−1.7)
*Women (596 558)*	123.7 (35.3)	123.4 (35.3)	−0.3 (−0.2)
Blood glucose			
*Total (1 632 013)*	101.5 (31.9)	104.6 (31.9)	3.2 (3.2)
*Men (681 115)*	106.6 (35.5)	109.8 (35.5)	3.2 (3.0)
*Women (950 898)*	97.8 (28.5)	100.9 (28.5)	3.1 (3.2)
HbA1c			
*Total (452 939)*	6.6 (1.3)	6.7 (1.3)	0.1 (1.9)
*Men (230 596)*	6.7 (1.3)	6.8 (1.3)	0.1 (1.9)
*Women (222 343)*	6.5 (1.2)	6.7 (1.2)	0.1 (1.9)
BMI			
*Total (862 821)*	28 (5.7)	28.3 (5.7)	0.3 (0.9)
*Men (376 328)*	28.1 (5.3)	28.3 (5.3)	0.1 (0.5)
*Women (486 493)*	28.0 (6.1)	28.3 (6.1)	0.4 (1.2)
SBP			
*Total (1 379 309)*	126.4 (16.4)	128.3 (16.4)	1.9 (1.5)
*Men (605 004)*	128.3 (16.0)	130.1 (16.0)	1.8 (1.4)
*Women (774 305)*	124.9 (16.5)	126.9 (16.5)	2.0 (1.6)
DBP			
*Total (1 379 362)*	74.5 (10.7)	75.6 (10.7)	1.2 (1.6)
*Men (605 024)*	75.2 (11.1)	76.4 (11.1)	1.2 (1.6)
*Women (774 338)*	73.8 (10.3)	75 (10.3)	1.2 (1.6)
Triglycerides			
*Total (1 194 695)*	131.3 (83.8)	134.9 (83.8)	3.6 (2.8)
*Men (525 700)*	141.8 (98.7)	143.3 (98.7)	1.6 (1.1)
*Women (668 995)*	123.0 (68.7)	128.3 (68.7)	5.3 (4.3)
REGICOR index			
*Total (250 992)*	4.8 (3.2)	5.1 (3.2)	0.3 (7.2)
*Men (122 508)*	5.9 (3.6)	6.5 (3.6)	0.6 (9.4)
*Women (128 484)*	3.7 (2.3)	3.8 (2.3)	0.1 (3.7)
Barthel index			
*Total (94 063)*	70.6 (28.5)	59.6 (28.5)	−11.1 (−15.7)
*Men (32 249)*	75.8 (27.6)	65.1 (27.6)	−10.6 (−14.1)
*Women (61 814)*	68.0 (28.5)	56.7 (28.5)	−11.3 (−16.6)

BMI – body mass index, DBP – diastolic blood pressure, HbA1c – fasting blood glucose/glycosylated haemoglobin, HDL-C – high-density lipoprotein cholesterol, LDL-C – low-density lipoprotein cholesterol, *REGICOR* – *Registre Gironí del cor* index, SBP – systolic blood pressure, SD – standard deviation, x̄ – mean

*Wilcoxon test for the difference of the medians was *P* < 0.001 for all comparisons.

Stratification by sex showed that pre-pandemic indicators were generally worse in men compared to women, excluding total cholesterol, LDL-C, and the Barthel index. However, almost all values deteriorated during the pandemic in both sexes, albeit with some differences. Specifically, the *REGICOR* index worsened more in men than in women (+9.4% in men vs +3.7% in women), while triglycerides (+1.1% in men vs +4.4% in women) and the Barthel index (−14.1% in men vs −16.6% in women) deteriorated more in women (**Table 1**).

### Repeated measurements analysis of clinical indicators before and during the pandemic according to major diagnoses and stratified by sex

#### Patients with HBP

The Barthel index (−15.7%), the *REGICOR* index (+6.1%), fasting blood glucose (+3.0%), and triglycerides (+2.2%) were the most affected indicators during the pandemic. The increase in the *REGICOR* index was more pronounced in men (+8.7%) than in women (+2.0%). Conversely, other factors worsened in women, either decreasing (as is the case with the Barthel index (−14.0% in men vs −16.5% in women) or increasing more (as we observed with triglyceride levels (+0.7% in men vs +3.7% in women)) (**Table 2**).

**Table 2:** Comparison of clinical indicators before and during the pandemic in the population with high blood pressure overall and stratified by sex

Clinical indicators (n)	Pre-pandemic, x̄ (SD)	Pandemic, x̄ (SD)	Difference (%)*
Cholesterol			
*Total (687 113)*	195.1 (41.7)	194.0 (41.7)	−1.1 (−0.6)
*Men (319 335)*	184.5 (40.9)	182.5 (40.9)	−2 (−1.1)
*Women (367 778)*	204.4 (40.1)	204 (40.1)	−0.4(−0.2)
HDL-C			
*Total (551 512)*	54.2 (14.1)	54.9 (14.1)	0.8 (1.4)
*Men (256 690)*	49.2 (12.4)	49.9 (12.4)	0.7 (1.5)
*Women (294 822)*	58.5 (14.1)	59.3 (14.1)	0.8 (1.3)
LDL-C			
*Total (550 995)*	115.6 (35.7)	113.2 (35.7)	−2.4 (−2.1)
*Men (256 320)*	108.8 (35)	105.9 (35)	−2.9 (−2.7)
*Women (294 675)*	121.5 (35.2)	119.6 (35.2)	−2 (−1.6)
Fasting blood glucose			
*Total (720 462)*	110.4 (35.9)	113.7 (35.9)	3.3 (3)
*Men (336 392)*	113.9 (37.9)	117 (37.9)	3.2 (2.8)
*Women (384 070)*	107.3 (33.9)	110.8 (33.9)	3.5 (3.3)
HbA1c			
*Total (304 588)*	6.7 (1.2)	6.8 (1.2)	0.1 (1.8)
*Men (156 690)*	6.7 (1.3)	6.8 (1.3)	0.1 (1.8)
*Women (147 898)*	6.7 (1.2)	6.8 (1.2)	0.1 (1.9)
BMI			
*Total (449 647)*	29.7 (5.2)	29.6 (5.2)	−0.1 (−0.3)
*Men (217 498)*	29.5 (4.7)	29.4 (4.7)	−0.1 (−0.4)
*Women (232 149)*	29.9 (5.7)	29.9 (5.7)	−0.1 (−0.2)
SBP			
*Total (658 250)*	133.7 (13.9)	134.2 (13.9)	0.5 (0.3)
*Men (310 817)*	134.2 (13.6)	134.5 (13.6)	0.3 (0.2)
*Women (347 433)*	133.3 (14.2)	133.9 (14.2)	0.6 (0.5)
DBP			
*Total (658 301)*	76.9 (10.3)	76.9 (10.3)	0.1 (0.1)
*Men (310 835)*	77.6 (10.5)	77.6 (10.5)	0 (0)
*Women (347 466)*	76.2 (10.1)	76.3 (10.1)	0.1 (0.2)
Triglycerides			
*Total (618 515)*	139.0 (83.5)	142.2 (83.5)	3.1 (2.2)
*Men (290 938)*	143.8 (95.4)	144.8 (95.4)	1 (0.7)
*Women (327 577)*	134.8 (71)	139.9 (71)	5 (3.7)
REGICOR index			
*Total (161 690)*	5.2 (3.3)	5.5 (3.3)	0.3 (6.1)
*Men (83 708)*	6.2 (3.7)	6.8 (3.7)	0.5 (8.7)
*Women (77 982)*	4 (2.4)	4.1 (2.4)	0.1 (2)
Barthel index			
*Total (76 313)*	71.6 (27.5)	60.4 (27.5)	−11.2 (−15.7)
*Men (24 900)*	77.3 (26.2)	66.4 (26.2)	−10.8 (−14)
*Women (51 413)*	68.8 (27.7)	57.4 (27.7)	−11.4 (−16.5)

BMI – body mass index, DBP – diastolic blood pressure, HbA1c – fasting blood glucose/glycosylated haemoglobin, HDL-C – high-density lipoprotein cholesterol, LDL-C – low-density lipoprotein cholesterol, *REGICOR* – *Registre Gironí del cor* index, SBP – systolic blood pressure, SD – standard deviation, x̄ – mean

*Wilcoxon test for the difference of the medians was *P* < 0.001 for all comparisons.

Clinical indicators of individuals without HBP worsened to a similar or greater extent than in those with HBP, with a notable increase in the *REGICOR* index (+9.5), particularly in men (+11.4%) compared to women (+7.1%) (Table S6 in the **Online Supplementary Document**).

Furthermore, almost all indicators worsened in patients diagnosed with HBP during the pandemic than in those diagnosed before this period, especially for the *REGICOR* index (+5.6% diagnosed before the pandemic vs +20.3% during the pandemic), reaching an increase of +23.6% for men (Tables S7–8 in the **Online Supplementary Document**).

#### Patients with HF

The most significantly deteriorated indicator during the pandemic was the Barthel index (15.5%), followed by the *REGICOR* index (+4.3%), fasting blood glucose (+2.8%), and triglyceride levels (+1.8%). The *REGICOR* index only worsened for men during the pandemic (+7.3% in men vs −1.6% in women), while other indicators deteriorated in women, including the Barthel index (−14.3% in men vs −16.2% in women), fasting blood glucose (+2.4% in men vs +3.1% in women), and triglyceride levels (+0.5% in men vs +2.9% in women) (**Table 3**).

**Table 3:** Comparison of clinical indicators before and during the pandemic in the population with heart failure overall and stratified by sex

Clinical indicators (n)	Pre-pandemic, x̄ (SD)	Pandemic, x̄ (SD)	Difference (%)*
Cholesterol			
*Total (136 466)*	184.4 (42.2)	181.2 (42.2)	−3.2 (−1.7)
*Men (62 247)*	172.3 (40)	168.4 (40)	−3.9 (−2.3)
*Women (74 219)*	194.5 (41.3)	192 (41.3)	−2.5 (−1.3)
HDL-C			
*Total (*108 139*)*	53.3 (14.4)	53.5 (14.4)	0.2 (0.4)
*Men (50 048)*	48.6 (12.8)	48.8 (12.8)	0.2 (0.5)
*Women (58 091)*	57.3 (14.5)	57.6 (14.5)	0.2 (0.4)
LDL-C			
*Total (107 989)*	106.8 (35.6)	103 (35.6)	−3.8 (−3.6)
*Men (49 943)*	99.2 (33.9)	94.8 (33.9)	−4.4 (−4.4)
*Women (58 046)*	113.4 (35.7)	110.1 (35.7)	−3.3 (−2.9)
Fasting blood glucose			
*Total (144 914)*	110.6 (36.9)	113.7 (36.9)	3 (2.8)
*Men (66 336)*	113.9 (38)	116.6 (38)	2.7 (2.4)
*Women (78 578)*	107.9 (35.7)	111.2 (35.7)	3.3 (3.1)
HbA1c			
*Total (65 125)*	6.7 (1.2)	6.8 (1.2)	0.1 (1.6)
*Men (32 955)*	6.7 (1.2)	6.8 (1.2)	0.1 (1.6)
*Women (32 170)*	29.8 (5.9)	29.6 (5.9)	−0.2 (−0.8)
BMI			
*Total (91 573)*	29.5 (5.5)	29.3 (5.5)	−0.3 (−0.8)
*Men (44 044)*	29.3 (4.9)	29 (4.9)	−0.3 (−0.9)
*Women (47 529)*	131.5 (15.6)	131.5 (15.6)	0 (0)
SBP			
*Total (135 535)*	131.3 (15.3)	131.2 (15.3)	−0.1 (−0.1)
*Men (62 934)*	131.1 (14.9)	130.9 (14.9)	−0.2 (−0.2)
*Women (72 601)*	72.8 (10.3)	72.9 (10.3)	0.1 (0.2)
DBP			
*Total (135 541)*	73.1 (10.5)	73.2 (10.5)	0.1 (0.1)
*Men (62 934)*	73.5 (10.7)	73.5 (10.7)	0 (0)
*Women (72 607)*	6.7 (1.2)	6.8 (1.2)	0.1 (1.6)
Triglycerides			
*Total (121 241)*	133.2 (75.7)	135.7 (75.7)	2.4 (1.8)
*Men (56 221)*	133.6 (82.5)	134.3 (82.5)	0.7 (0.5)
*Women (65 020)*	132.9 (69.3)	136.8 (69.3)	3.9 (2.9)
REGICOR index			
*Total (20 339)*	5.4 (3.5)	5.6 (3.5)	0.2 (4.3)
*Men (11 597)*	6.3 (3.8)	6.8 (3.8)	0.5 (7.3)
*Women (8742)*	4.1 (2.6)	4.1 (2.6)	−0.1 (−1.6)
Barthel index			
*Total (3 124)*	73 (25.4)	61.7 (25.4)	−11.3 (−15.5)
*Men (10 662)*	79.1 (24.1)	67.8 (24.1)	−11.3 (−14.3)
*Women (20 462)*	69.9 (25.5)	58.6 (25.5)	−11.3 (−16.2)

BMI – body mass index, DBP – diastolic blood pressure, HbA1c – fasting blood glucose/glycosylated haemoglobin, HDL-C – high-density lipoprotein cholesterol, LDL-C – low-density lipoprotein cholesterol, *REGICOR* – *Registre Gironí del cor* index, SBP – systolic blood pressure, SD – standard deviation, x̄ – mean

*For the overall analyses, the Wilcoxon test for the difference of the medians was *P* < 0.001 for all comparisons except for SBP (*P*-value not significant), and DBP (*P* < 0.05). For men, the Wilcoxon test for the difference of the medians was *P* < 0.001 for all comparisons, except for SBP (*P* < 0.05), DBP (*P*-value not significant), and triglycerides (*P* < 0.01). For women, the Wilcoxon test of the difference of the medians was *P* < 0.001 for all comparisons except for SBP (*P*-value not significant) and DBP (*P* < 0.01).

For individuals without HF, almost all indicators worsened, most notably the Barthel index (−15.8%) and the *REGICOR* index (+7.4%), with the latter worsening to a greater extent than in HF patients. Unlike for HF patients, the *REGICOR* index worsened in both men and women (+9.7% in men vs +4.2% in women) (Table S9 in the **Online Supplementary Document**). Overall, HF patients diagnosed during the pandemic showed a better evolution of the indicators than those diagnosed before the pandemic, except for the Barthel index (−15.0% diagnosed before the pandemic vs −19.7% during the pandemic). The evolution of the *REGICOR* index worsened for men diagnosed with HF before the pandemic (+7.8% in men vs −1.7% in women) and during the pandemic (+3.5% in men vs −1.0% in women) (Tables S10–11 in the **Online Supplementary Document**).

#### Patients with T2D

In patients with T2D, the Barthel index deteriorated the most during the pandemic (−15.1%), followed by the *REGICOR* index (+7.7%), fasting blood glucose (+3.3%), and HbA1c (+2.0%). Like other diagnoses, the *REGICOR* index declined more in men (+9.5%) than in women (+4.5%). Conversely, the Barthel index decreased more in women (−13.7%) than men (−16.0%) (**Table 4**).

**Table 4:** Comparison of clinical indicators before and during the pandemic in the population with diabetes overall and stratified by sex

Clinical indicators (n)	Pre-pandemic, x̄ (SD)	Pandemic, x̄ (SD)	Difference (%)*
Cholesterol			
*Total (435 577)*	188.3 (41.8)	186.9 (41.8)	−1.4 (−0.7)
*Men (222 348)*	179.4 (41.1)	177.1 (41.1)	−2.2 (−1.2)
*Women (213 229)*	197.6 (40.6)	197.2 (40.6)	−0.4 (−0.2)
HDL-C			
*Total (355 522)*	51.3 (13.3)	52.1 (13.3)	0.8 (1.6)
*Men (183 332)*	47.5 (11.9)	48.3 (11.9)	0.8 (1.6)
*Women (172 190)*	55.3 (13.4)	56.2 (13.4)	0.9 (1.6)
LDL-C			
*Total (355 024)*	109.1 (35.2)	106.2 (35.2)	−2.8 (−2.6)
*Men (182 953)*	103.3 (34.3)	100.0 (34.3)	−3.2 (−3.1)
*Women (172 071)*	115.2 (35.2)	112.8 (35.2)	−2.4 (−2.1)
Fasting blood glucose			
*Total (456 950)*	130.5 (44)	134.8 (44.0)	4.3 (3.3)
*Men (232 854)*	134.3 (45.2)	138.3 (45.2)	4.0 (3.0)
*Women (224 096)*	126.4 (42.3)	131.1 (42.3)	4.7 (3.7)
HbA1c			
*Total (351 981)*	6.9 (1.3)	7.0 (1.3)	0.1 (2.0)
*Men (186 651)*	6.9 (1.3)	7.1 (1.3)	0.1 (2.0)
*Women (165 330)*	6.8 (1.2)	7.0 (1.2)	0.1 (2.1)
BMI			
*Total (306 620)*	30.2 (5.3)	30.0 (5.3)	−0.2 (−0.6)
*Men (160 898)*	29.7 (4.7)	29.5 (4.7)	−0.2 (−0.8)
*Women (145 722)*	30.7 (5.8)	30.6 (5.8)	−0.1 (−0.3)
SBP			
*Total (396 965)*	132.5 (14.0)	133.2 (14)	0.7 (0.5)
*Men (204 839)*	133.3 (13.4)	133.8 (13.4)	0.5 (0.4)
*Women (192 126)*	131.7 (14.5)	132.6 (14.5)	0.9 (0.7)
DBP			
*Total (396 991)*	75.9 (9.9)	76.1 (9.9)	0.2 (0.3)
*Men (204 849)*	76.5 (10)	76.6 (10)	0.1 (0.1)
*Women (192 142)*	75.3 (9.7)	75.6 (9.7)	0.3 (0.4)
Triglycerides			
*Total (400 199)*	152.7 (99.0)	155.7 (99.0)	3.0 (2.0)
*Men (207 744)*	156.0 (110.7)	156.9 (110.7)	0.9 (0.6)
*Women (192 455)*	149.1 (84.4)	154.4 (84.4)	5.3 (3.5)
REGICOR index			
*Total (116 382)*	6.1 (3.6)	6.5 (3.6)	0.5 (7.7)
*Men (66 348)*	6.9 (4)	7.5 (4)	0.7 (9.5)
*Women (50 034)*	5.0 (2.8)	5.2 (2.8)	0.2 (4.5)
Barthel index			
*Total (42 215)*	72.6 (27.2)	61.7 (27.2)	−10.9 (−15.1)
*Men (16 566)*	77.5 (26.2)	66.9 (26.2)	−10.6 (−13.7)
*Women (25 649)*	69.4 (27.3)	58.3 (27.3)	−11.1 (−16.0)

BMI – body mass index, DBP – diastolic blood pressure, HbA1c – fasting blood glucose/glycosylated haemoglobin, HDL-C – high-density lipoprotein cholesterol, LDL-C – low-density lipoprotein cholesterol, *REGICOR* – *Registre Gironí del cor* index, SBP – systolic blood pressure, SD – standard deviation, x̄ – mean

*Wilcoxon test of the difference of the medians was *P* < 0.001 for all comparisons.

The evolution of indicators during the pandemic was generally worse in patients without T2D than in patients with T2D, except for the *REGICOR* index, fasting blood glucose, and HbA1c (Table S12 in the **Online Supplementary Document**). Moreover, patients with T2D diagnosed during the pandemic had a significantly worse evolution for fasting blood glucose (+2.7% diagnosed before the pandemic vs +13.7% during the pandemic), HbA1c (+1.9% diagnosed before the pandemic vs +7.4% during the pandemic), and *REGICOR* index (+6.8% diagnosed before the pandemic vs +27.8% during the pandemic) (Tables S13–14 in the **Online Supplementary Document**).

## DISCUSSION

We conducted an extensive analysis to assess the repercussions of the COVID-19 pandemic on clinical indicators using longitudinal data from *SIDIAP*, the largest primary care database in Catalonia, encompassing the description of mortality and prevalent diagnoses, among other variables. We observed a negative impact on clinical indicators in the pandemic period, particularly on cardiometabolic risk. Sex differences were also evident, with men displaying higher cardiovascular risk and women experiencing greater functional decline during the pandemic.

The active population in the public health system decreased by 1.4% during the pandemic when compared to the last follow-up date of the study. This decline was present due to increased mortality rates, both directly and indirectly related to COVID-19, as well as higher transfer rates among the younger population (aged 25 to 44 years) likely due to them returning to their places of origin. Mean mortality rates showed the most notable increase during the first wave (from 0.07% before the pandemic to 0.17% in the first wave), followed by a decline in the second (0.09%) and third (0.11%) waves. Accordingly, previous research carried out on the Spanish population showed a substantial decrease in excess mortality from the first (44 583 deaths) to the second waves (24 373 deaths), with a further decline in the third wave (14 040 deaths) [19]. This drop in mortality may be attributed to improved pandemic control measures and a potential harvesting effect after an initial surge in deaths among the elderly [19]. In our analysis, mortality disproportionately affected the elderly, with the highest impact observed in patients over 85 years (increasing from mean rates of 1.0% to 2.5%). This was an expected result, as patients ≥80 years old were found to have the highest odds of death from COVID-19 compared to younger ones (odds ratio (OR) = 28.5; 95% confidence interval (CI) = 19.9, 40.8) [20]. Additionally, lower-income individuals had higher mean mortality rates, particularly evident in the first wave, while the disparity between low- and high-income areas became more pronounced in the third wave (mean rates of 0.15% for the 4th quartile of the *MEDEA* deprivation index and approximately 0.10% for other quartiles). These findings align with a previous study that identified higher COVID-19 mortality rates in Barcelona neighbourhoods with lower socioeconomic status (15.9%) compared to those with higher status (10.0%) [21] and might indicate a potential worsening of social inequalities during the pandemic.

We also found a decline in the period prevalence of chronic diseases. Speficially, HF patients showed the greatest decrease (−26.7%), followed by HBP (−15.1%) and T2D (−14.6%) patients. This decrease in period prevalence could be attributed to a decrease in new diagnoses, likely due to delayed diagnosis of chronic diseases during the pandemic. For instance, a previous report indicated an 11% lower incidence of chronic diseases in 2020 compared to 2019 [22], and another study showed a lower incidence than expected during the first wave of the pandemic for hypertension (−25.5%), diabetes (−24.0%), and heart failure (−13.3%) [12,23]. The increased risk of COVID-19 mortality among individuals with chronic conditions may have also contributed to the diminished prevalence in these populations [24].

Our primary objective was to compare the repeated measurements of key clinical indicators before and during the pandemic. Our findings revealed a deterioration in most indicators during the pandemic period, with the *REGICOR* index showing a significant 7.2% increase, indicating aggravated cardiovascular risk for the population. Other clinical indicators such as total cholesterol, triglycerides, fasting blood glucose, and blood pressure also worsened. These findings are consistent with a previous study conducted in Spain [25] which also reported deteriorated parameters related to cardiometabolic health during lockdown, including HDL-C, waist circumference, triglycerides, blood glucose, and blood pressure. The worsening of these indicators could be attributed to unhealthy lifestyle changes and reduced monitoring and control of cardiometabolic parameters during the pandemic [11,25]. The Barthel index (a measure of functional independence) decreased significantly by 15.7%, which could be attributed to severe COVID-19 infection and confinement, leading to both physical and cognitive decline and a reduction in the ability to perform daily living activities, particularly among the elderly population [26,27].

Most clinical indicators worsened during the pandemic for both sexes. The *REGICOR* index showed more significant deterioration in men (+9.4%) than in women (+3.7%), likely due to worse baseline values [28]. In contrast, the Barthel index declined more in women (−16.6%) than in men (−14.1%), possibly due to a larger elderly female population and a greater physical and mental impact of the pandemic on women [29]. Despite patients with chronic conditions (HF, HBP, and T2D) having worse pre-pandemic indicators, these worsened similarly or to a greater extent in individuals without these conditions. This suggests that the absence of chronic diseases might lead to the underestimation of cardiovascular risk, as previously reported [30], and poorer control of cardiometabolic factors. Notably, patients diagnosed with chronic conditions during the pandemic experienced a significant decline in clinical indicators, particularly the *REGICOR* index. This effect was particularly pronounced in patients with HBP (+5.6% diagnosed before the pandemic vs +20.3% diagnosed during the pandemic) and T2D (+6.8% diagnosed before the pandemic vs +27.8% diagnosed during the pandemic), highlighting the negative impact of limited access to appropriate care on cardiometabolic disease indicators during the pandemic.

Our analysis has some limitations that need to be acknowledged. First, our exploratory design does not allow us to confirm a causal relationship between the pandemic and worsening clinical indicators, but only temporary trends before and during the pandemic. Moreover, although many parameters significantly worsened during the pandemic, we cannot determine the actual impact on population health as some indicators remained within the normal range. Furthermore, collection of our patients’ data was based on medical criteria rather than random selection, limiting data available to patients with poorer health or suspected illness. In addition, some subgroup analyses, particularly for the Barthel index, had a limited number of individuals, potentially affecting the robustness of conclusions. Finally, while our analysis focuses on the region of Catalonia, we believe it provides a comprehensive view of the effects of COVID-19 on population health, considering the wide range of clinical indicators analysed (laboratory tests, vital signs, indexes) that are routinely recorded at the primary level in any settings.

## CONCLUSIONS

To our knowledge, this is the first study to use longitudinal data in assessing the repercussions of the COVID-19 pandemic on population health considering a wide range of clinical indicators, alongside socioeconomic and sex determinants. Our analysis demonstrates a worsening in clinical indicators during the pandemic, particularly in cardiometabolic factors and cardiovascular risk. Additionally, we observed significant disparities in mortality rates, notably among the elderly and low-income populations. Our findings underscore the significance of ensuring continuous care to individuals with chronic conditions and addressing gender and socioeconomic disparities that have been exacerbated during the pandemic.

## Additional material


Online Supplementary Document.
