# Supplementary material for: Impacts of COVID-19 on clinical indicators and mortality in patients with chronic conditions in Catalonia, Spain: A retrospective population-based cohort study
Source: J Glob Health. 2024 Jun 21;14:05020. doi: 10.7189/jogh.14.05020 (PMC11189023; doi:10.7189/jogh.14.05020)
Supplement: Online Supplementary Document. [file jogh-14-05020-s001.pdf]

Online Supplementary Document

Moreno M, Vidal-Alaball J, Saez M, Barceló M. Impacts of COVID-19 on clinical indicators and mortality in Catalan patients, Spain, with chronic conditions: A retrospective population-based cohort study.

Supplementary Tables Index

Table S1. RECORD checklist.

Table S2. Description of variables extracted from the SIDIAP database.

Table S3. Description of variables estimated from other sources.

Table S4. Frequencies and proportions on demographics and prevalent chronic diseases overall and grouped by the pre- and pandemic periods (whole SIDIAP population).

Table S5. Number of deaths among those diagnosed with some chronic conditions and average annual mortality rate (per 1,000 people diagnosed.

Table S6. Comparison of health indicators before and during the pandemic in the population without HBP and stratified by sex.

Table S7. Comparison of health indicators in the population with HBP diagnosed before the pandemic and stratified by sex.

Table S8. Comparison of health indicators in the population with HBP diagnosed during the pandemic and stratified by sex.

Table S9. Comparison of health indicators before and during the pandemic in the population without HF and stratified by sex.

Table S10. Comparison of health indicators in the population with HF diagnosed before the pandemic and stratified by sex.

Table S11. Comparison of health indicators in the population with HF diagnosed during the pandemic and stratified by sex.

Table S12. Comparison of health indicators before and during the pandemic in the population without T2D and stratified by sex.

Table S13. Comparison of health indicators in the population with T2D diagnosed before the pandemic and stratified by sex.

Table S14. Comparison of health indicators in the population with T2D diagnosed during the pandemic and stratified by sex.

Supplementary Figures Index

Figure S1. Flow chart of the study cohort.

Figure S2. Representation of deaths from January 2018 to June 2021 (before and during the pandemic) for the total population (A); the population stratified by age groups (B); the population stratified by pharmaceutical co-payment (C); and the population stratified by MEDEA index quartiles (D). In (A), the line represents the crude death rate on the left axis, the bars represent death counts on the right axis for the total population, and the lines in (B-D) represent death rates.

Figure S3. Representation of the transfer rate from January 2018 to June 2021 (before and during the pandemic). For the total population (A); the population stratified by age groups (B).

Table S1. RECORD checklist[1]

Section and Topic	Item #	STROBE items	Location in manuscript where items are reported	RECORD items	Location in manuscript where items are reported
**TITLE AND ABSTRACT**					
Title	1	(a) Indicate the study's design with a commonly used term in the title or the abstract (b) Provide in the abstract an informative and balanced summary of what was done and what was found	Page X; Title page and abstract	RECORD 1.1: The type of data used should be specified in the title or abstract. When possible, the name of the databases used should be included.<br>RECORD 1.2: If applicable, the geographic region and timeframe within which the study took place should be reported in the title or abstract.<br>RECORD 1.3: If linkage between databases was conducted for the study, this should be clearly stated in the title or abstract.	Page 1; Title page<br>Page 3: Abstract<br>Not applicable
**INTRODUCTION**					
Background rationale	2	Explain the scientific background and rationale for the investigation being reported	Page X; Introduction		Pages 7-8; Background
Objectives	3	State specific objectives, including any prespecified hypotheses	Page X; Introduction		Page 8; Background
**METHODS**					
Study Design	4	Present key elements of study design early in the paper	Page X; Methods, Study Design, Data Source, and Population		Page 9, Methods, Study Desing, Data Source and Population
Setting	5	Describe the setting, locations, and relevant dates, including periods of recruitment, exposure, follow-up, and data collection	Page X; Methods, Study Design, Data Source, and Population		Pages 9-10; Methods, Study Desing, Data Source and Population
Participants	6	(a) Cohort study<br>- Give the eligibility criteria, and the sources and methods of selection of participants. Describe methods of follow-up<br>Case-control study<br>- Give the eligibility criteria, and the sources and methods of case ascertainment and control selection. Give the rationale for the choice of cases and controls<br>Cross-sectional study<br>- Give the eligibility criteria, and the sources and methods of selection of participants<br>(b) Cohort study<br>- For matched studies, give matching criteria	Page X; Methods, Study Design, Data Source, and Population	RECORD 6.1: The methods of study population selection (such as codes or algorithms used to identify subjects) should be listed in detail. If this is not possible, an explanation should be provided.<br>RECORD 6.2: Any validation studies of the codes or algorithms used to select the population should be referenced. If validation was conducted for this study and not published elsewhere, detailed methods and results should be provided.<br>RECORD 6.3: If the study involved linkage of databases, consider use of a flow diagram or other graphical display to demonstrate the data linkage process, including the number of individuals with linked data at each stage.	Page 9; Methods, Study Design, Data Source, and Population.<br>Table S2.<br>Not applicable.<br>Reference 14 on the same database (SIDIAP):<br>Table S2 (diagnostic codes)<br>Not applicable

Section and Topic	Item #	STROBE items	Location in manuscript where items are reported	RECORD items	Location in manuscript where items are reported
		and number of exposed and unexposed<br>Case-control study<br>- For matched studies, give matching criteria and the number of controls per case			
Variables	7	Clearly define all outcomes, exposures, predictors, potential confounders, and effect modifiers. Give diagnostic criteria, if applicable.	Page X; Methods, Variables and Supplementary Tables 2 and 3	RECORD 7.1: A complete list of codes and algorithms used to classify exposures, outcomes, confounders, and effect modifiers should be provided. If these cannot be reported, an explanation should be provided.	Pages 10-11. Methods, Variables<br>Table S2<br>Table S3
Data sources/ measurement	8	For each variable of interest, give sources of data and details of methods of assessment (measurement). Describe comparability of assessment methods if there is more than one group.	Page X; Methods, Variables and Supplementary Tables 2 and 3		Pages 10-11. Methods, Variables<br>Table S2<br>Table S3
Bias	9	Describe any efforts to address potential sources of bias.	-		--
Study size assessment	10	Explain how the study size was arrived at	-		Page 11. Methods, Statistical analysis
Quantitative variables	11	Explain how quantitative variables were handled in the analyses. If applicable, describe which groupings were chosen, and why.	Page X; Methods, Statistical Analysis		Page 11. Methods, Statistical analysis
Synthesis methods	12a	Describe all statistical methods, including those used to control for confounding	Page X; Methods, Statistical Analysis		Pages 11-12. Methods, Statistical analysis
	12b	Describe any methods used to examine subgroups and interactions.	Page X; Methods, Statistical Analysis		Pages 11-12. Methods, Statistical analysis
	12c	Explain how missing data were addressed	-		Pages 11-12. Methods, Statistical analysis
	12d	Cohort study - If applicable, explain how loss to follow - up was addressed Case -control study - If applicable, explain how matching of cases and controls was addressed Cross - sectional study - If applicable, describe analytical methods taking account of sampling strategy	-		Pages 11-12. Methods, Statistical analysis
	12e	Describe any sensitivity analyses			Not applicable
Data access and cleaning methods				RECORD 12.1: Authors should describe the extent to which the investigators had access to the database population used to create the study population.	Page 12. Methods. Data access.

Section and Topic	Item #	STROBE items	Location in manuscript where items are reported	RECORD items	Location in manuscript where items are reported
				RECORD 12.2: Authors should provide information on the data cleaning methods used in the study	Not applicable
Linkage			-	RECORD 12.3: State whether the study included person -level, institutional -level, or other data linkage across two or more databases. The methods of linkage and methods of linkage quality evaluation should be provided.	Not applicable
**RESULTS**					
Participants	13a	Report the numbers of individuals at each stage of the study (e.g., numbers potentially eligible, examined for eligibility, confirmed eligible, included in the study, completing follow -up, and analysed)	Page X; Results, Cohort Description: Sociodemographics and Most Prevalent Diagnoses	RECORD 13.1: Describe in detail the selection of the persons included in the study (i.e., study population selection) including filtering based on data quality, data availability and linkage. The selection of included persons can be described in the text and/or by means of the study flow diagram.	Pages 12-13. Results. Cohort Description and Most Prevalent Diagnoses.<br>Figure 1 (flow diagram)
	13b	Give reasons for non - participation at each stage.	Page X; Results, Cohort Description: Sociodemographics and Most Prevalent Diagnoses		Figure 1 (flow diagram)
	13c	Consider use of a flow diagram	Figure 1		Figure 1 (flow diagram)
Descriptive data	14a	Give characteristics of study participants (e.g., demographic, clinical, social) and information on exposures and potential confounders.	Page X; Results, Cohort Description: Sociodemographics and Most Prevalent Diagnoses		Pages 12-13. Results. Cohort Description and Most Prevalent Diagnoses.<br>Table 1
	14b	Indicate the number of participants with missing data for each variable of interest	Table 1		Table 1 (indicates the n with available data.
	14c	Cohort study - summarise follow -up time (e.g., average and total amount)	-		Not applicable
Outcome data	15	Cohort study - Report numbers of outcome events or summary measures over time Case -control study - Report numbers in each exposure category, or summary measures of exposure Cross -sectional study - Report numbers of outcome events or summary measures	Page X; Results		Pages 13-17. Results<br>Tables 2, 3, 4, and 5. Figure 2.<br>Tables S4 to 12.<br>Figure S1.
Main results	16a	Give unadjusted estimates and, if applicable, confounder - adjusted estimates and their precision (e.g., 95% confidence interval). Make clear which confounders were adjusted for and why they were included.	Page X; Results		Pages 13-17. Results<br>Tables 2, 3, 4, and 5. Figure 2.<br>Tables S4 to S12.<br>Figure 1.
	16b	Report category boundaries when continuous variables were categorized	Page X; Results		Not applicable for outcome measures.
	16c	If relevant, consider translating estimates of relative risk into absolute risk for a meaningful time period	-		

Section and Topic	Item #	STROBE items	Location in manuscript where items are reported	RECORD items	Location in manuscript where items are reported
Other analyses	17	Report other analyses done — e.g., analyses of subgroups and interactions, and sensitivity analyses	-		Not applicable
**DISCUSSION**					
Key results	18	Summarise key results with reference to study objectives	Page X; Discussion		Page 17, Discussion.
Limitations	19	Discuss limitations of the study, taking into account sources of potential bias or imprecision. Discuss both direction and magnitude of any potential bias	Page X; Discussion	RECORD 19.1: Discuss the implications of using data that were not created or collected to answer the specific research question(s). Include discussion of misclassification bias, unmeasured confounding, missing data, and changing eligibility over time, as they pertain to the study being reported	Page 20, Discussion.
Interpretation	20	Give a cautious overall interpretation of results considering objectives, limitations, multiplicity of analyses, results from similar studies, and other relevant evidence	Page X; Discussion		Pages 17-20, Discussion.
Generalisability	21	Discuss the generalisability (external validity) of the study results	Page X; Discussion		Pages 20-21, Discussion
**OTHER INFORMATION**					
Funding	22	Give the source of funding and the role of the funders for the present study and, if applicable, for the original study on which the present article is based	Page X; Role of the Funding Source		Page 12. Methods. Role of the funding source.
Accessibility of protocol, raw data, and programming code		Declare any competing interests of review authors.	Page X; Declaration of interest	RECORD 22.1: Authors should provide information on how to access any supplemental information such as the study protocol, raw data, or programming code.	Page 23. Data sharing.

Table S2. Description of variables extracted from the SIDIAP database[2]

Variables extracted from the SIDIAP database			
**SIDIAP status variables**	**SIDIAP code**	**Description**	**Categories/units**
Status in SIDIAP	situacio	Patient's status in SIDIAP as of 12/31/2021	Active, transferred (outside Catalonia's public health system), deceased
Date of SIDIAP entry	entrada	Date of patient entry into SIDIAP cohort	YYYYMMDD
Date of SIDIAP exit	Sortida	Date of patient's death or transfer	D (death)/T (transfer)/YYYYMMDD
**Sociodemographic variables**		**Description**	**Categories/units**
Sex	sexe	Patient's sex	Female, male
Date of birth	dnaix	Date of birth	YYYYMMDD
Region	provincia	Patient's region of residence	Girona, Lleida, Tarragona, Barcelona
Urbanicity	ruralitat2011	Urbanicity of the area where the patient lives, according to 2011 census sections	Rural, urban
Rurality	medea_centre	Type of rurality of the basic health area (ABS, acronym in Catalan)	Rural (Both a population density <100 inhabitants/Km2 and less than 7.500 inhabitants), semi-rural (Adherence to only one of the conditions that define rural category), and semi-urban (Both a population density >100 inhabitants/Km2 and between 7,500 and 10,000 inhabitants)
MEDEA deprivation index	medea_centre	Composite index of the socioeconomic level of the center (for urban centers) where the patient is assigned on the SIDIAP.	Quartiles (Q1, Q2, Q3, Q4); higher values of the indicator (quartile) show greater socioeconomic deprivation
Prescription cost-sharing contribution	apo_far	Proportion of patient out-of-pocket contribution for prescription cost-sharing	Exempt from contribution, 10% contribution, 40% contribution, 50% contribution, 60% contribution, pharmacy excluded
Maximum monthly prescription cost-sharing	apo_far_maxm	Maximum monthly out-of-pocket contribution for prescription cost-sharing	No monthly contribution limit, monthly contribution limit of €8.23, monthly contribution limit of €18.52, monthly contribution limit of €61.75
Annual income	nsi_far	Based on individual socioeconomic pharmacy contribution that serves as a proxy for annual income	Exempted from payment, <€18,000, between €18,001 and €100,000, >€100,000
**Most prevalent chronic disease**		**Description**	**Categories/units**
T2D	dm	Presence of T2D according to the ICD-10 code and registration date	ICD-10 code E11; Yes/No; YYYYMMDD
HBP	hta	Presence of HBP according to the ICD-10 code and registration date	ICD-10 code I10; Yes/No; YYYYMMDD
HF		Presence of HF according to the ICD-10 code and registration date	ICD-10 codes I20-I25; I50-I52; Yes/No; YYYYMMDD

Smoking status	val	Patient's smoking status and registration date	Current smokers, former smokers, non-smokers; YYYYMMDD
**Clinical indicators**		**Description**	**Categories/units**
SBP	pas	SBP value and registration date	mmHg (normal values <140 mmHg); YYYYMMDD
DBP	pad	DBP value and registration date	mmHg (normal values <90 mmHg); YYYYMMDD
BMI	IMC	BMI value and registration date	kg/m² (overweight between 25 kg/m^2 and 30 kg/m^2); YYYYMMDD
Total cholesterol	cT	Total cholesterol value and registration date	mg/dL (normal values of 125 to 200 mg/dL); YYYYMMDD
HDL-C	cHDL	HDL-C value and registration date	mg/dL (normal values of >40 mg/dL in males and >50 mg/dL in females of the same age range); YYYYMMDD
LDL-C	cLDL	LDL-C value and registration date	mg/dL (normal values <100 mg/dL); YYYYMMDD
Triglycerides	TG	Triglycerides value and registration date	mg/dL (normal values <150 mmHg); YYYYMMDD
Fasting blood glucose	FG	Fasting blood glucose value and registration date	mg/dL (normal values of 50 to 70 mg/dL; prediabetes values of 100 mg/dL to 125 mg/dL); YYYYMMDD
HbA1c	HbA1c	HbA1c value and registration date	% (normal values <5.7%; prediabetes values from 5.7% to 6.4%; diabetes values >6.4%); YYYYMMDD
Geriatric assessment (Barthel index)[34]	Barthel	Ordinal scale used to measure performance in activities of daily living; value and registration date	Scale from 0 to 100 (scores of 0-20 indicate “total” dependency, 21-60 indicate “severe” dependency, 61-90 indicate “moderate” dependency, and 91-99 indicate “slight” dependency); YYYYMMDD
REGICOR index[56]	REGICOR	Ten-year risk of the occurrence of a cardiovascular event (fatal myocardial infarction or not, silent myocardial infarction and angina pectoris); value and registration date	% (low risk <5%; moderate risk between 5% and 9.9%); YYYYMMDD

Abbreviations: BMI, body mass index; DBP, diastolic blood pressure; HbA1c, glycosylated hemoglobin; HBP, high blood pressure; HDL-C, high-density lipoprotein cholesterol; HF, heart failure; ICD-10, 10th revision of the International Statistical Classification of Diseases and Related Health Problems codes; LDL-C, low-density lipoprotein cholesterol; REGICOR, Registre Gironí del Cor (Girona Heart Registry); SBP, systolic blood pressure; T2D, type 2 diabetes mellitus; YYYYMMDD, year, month and day.

Table S3. Description of variables estimated from other variables.

**Variables estimated from other variables**			
**Variable**	**Description**	**Estimation**	**Categories/units**
Net income	Average net income weighted by population density	Patients' average income values for the years 2015 to 2019 observed in the Spanish census, INE[7]weighted by population density in each basic health zone in Catalonia[8]	Quartiles (Q1, Q2, Q3, Q4); higher values of the indicator (quartiles) show greater socioeconomic deprivation.
Gini index	Index for the degree of inequality in the distribution of income among different regions	Patients' average income values for the years 2015 to 2019 observed in the Spanish census, INE[7] weighted by population density in each basic health zone in Catalonia[8,9]	Quartiles (Q1, Q2, Q3, Q4); higher values of the indicator (quartiles) show greater socioeconomic deprivation.
Income category	Based on the pharmacy co-payment variable, which is a cost-sharing arrangement between a patient and a government health program. Serves as a proxy for income category.	The combined variable of the Prescription cost-sharing contribution (apo_far) and Maximum monthly prescription cost-sharing (apo_far_maxm)	Groups exempt from contribution (0%); pensioners (no contribution limit); pensioners - income <€18,000 (up to €8/month); pensioners - income range €18,000 - €100,000 (up to €18/m); workers - income <€18,000 (40%); workers - income range €18,000 - €100,000 (50%); workers - income >€100,000 (60%); pensioners - income >€100,000 (up to €61/month); others - income range €18,000 - €100,000 (excluded)

Abbreviations: INE, *Instituto Nacional de Estadística* (Spanish Statistical Office).

Table S4. Frequencies and proportions on demographics and prevalent chronic diseases overall and grouped by the pre- and pandemic periods (whole SIDIAP population)

Variables	All periods	Pre-pandemic period	Pandemic period
**Sex, *n (%)***	6,301,095	6,189,545 (98·2)	5,784,283 (91·8)
Female	3,220,037 (51·1)	3,163,543 (51·1)	2,956,387 (51·1)
Male	3,081,058 (48·9)	3,026,002 (48·9)	2,827,896 (48·9)
**Age (grouped), *n (%)***	6,301,095	6,189,545 (98·2)	5,784,283 (91·8)
0-15	930,672 (14·8)	876338 (14·2)	885,975 (15·3)
16-24	558,713 (8·9)	548,517 (8·9)	531,125 (9·2)
25-34	729,598 (11·6)	707,899 (11·4)	666,406 (11·5)
35-44	928,524 (14·7)	916,931 (14·8)	867,442 (15)
45-54	958,205 (15·2)	952,135 (15·4)	916,483 (15·8)
55-64	769,904 (12·2)	766,291 (12·4)	732,272 (12·7)
65-74	611,587 (9·7)	609,397 (9·8)	565,964 (9·8)
75-84	459,867 (7·3)	458,766 (7·4)	387,540 (6·7)
>85	354,025 (5·6)	353,271 (5·7)	231,076 (4·0)
**Region, *n (%)***	6,288,720	6,177,393 (98·2)	5,781,620 (91·9)
Girona	561,523 (8·9)	552,988 (8·9)	513,803 (8·9)
Lleida	424,368 (6·7)	415,813 (6·7)	390,340 (6·8)
Tarragona	577,154 (9·2)	567,302 (9·2)	526,429 (9·1)
Barcelona	4,725,675 (75·0)	4,641,290 (75·0)	4,351,048 (75·2)
Missing	12,375 (0·2)	12,152 (0·2)	2663 (0)
**Urbanicity, *n (%)***	5,720,113	5,612,592 (98·1)	5,382,642 (94·1)
Rural	356,914 (5·7)	351,558 (5·7)	332,112 (5·7)
Urban	5,363,199 (85·1)	5,261,034 (85)	5,050,530 (87·3)
Missing	580,982 (9·2)	576,953 (9·3)	401,641 (6·9)
**Rurality, *n (%)***	1,532,950	1,509,476 (98·5)	1,412,191 (92.1)
Rural	408,236 (26.6)	402,404 (26.7)	372,927 (26.4)
Semi-rural	365,156 (23.8)	357,552 (23.7)	336,206 (23.8)
Semi-urban	759,558 (49.5)	749,520 (49.7)	703,058 (49.8)

**MEDEA deprivation index, *n (%)***	**4,768,141**	4,680,065 (98.2)	4,372,092 (91.7)
Urban, very high socioeconomic level	1,347,269 (28.3)	1,319,555 (28.2)	1,224,573 (28.0)
Urban, high socioeconomic level	944,536 (19.8)	930,177 (19.9)	872,145 (19.9)
Urban, low socioeconomic level	1,290,901 (27.1)	1,269,402 (27.1)	1,187,841 (27.2)
Urban, very low socioeconomic level	1,185,435 (24.9)	1,160,931 (24.8)	1,087,533 (24.9)
Missing	4 (0)	4 (0)	0 (0)
**Income (quartile), *n (%)***	6,136,891	6,037,484 (98·4)	5,621,787 (91·6)
1st	1,668,735 (26·5)	1,641,710 (26·5)	1,526,304 (26·4)
2nd	1,209,767 (19·2)	1,190,726 (19·2)	1,113,404 (19·2)
3rd	1,510,145 (24·0)	1,487,707 (24)	1,387,471 (24)
4th	1,748,244 (27·8)	1,717,341 (27·8)	1,594,608 (27·6)
Missing	164,204 (2·6)	152,061 (2·5)	162,496 (2·8)
**Gini index (quartile), *n (%)***	6,136,891	6,037,484 (98·4)	5,621,787 (91·6)
1st	1,341,498 (21·3)	1,322,113 (21·4)	1,238,275 (21·4)
2nd	1,543,176 (24·5)	1,520,846 (24·6)	1,419,860 (24·6)
3rd	1,709,588 (27·1)	1,681,640 (27·2)	1,562,832 (27)
4th	1,542,629 (24·5)	1,512,885 (24·4)	1,400,820 (24·2)
Missing	164,204 (2·6)	152,061 (2·5)	162,496 (2·8)
**Annual income (proxy), *n (%)***	6,301,095	6,189,545 (98·2)	5,784,283 (91·8)
Not specified	609,899 (9·7)	552,545 (8·9)	448,685 (7·8)
Exempt	540,299 (8·6)	539,007 (8·7)	522,617 (9)
<€18·000	3,160,231 (50·1)	3,116,560 (50·4)	2,877,319 (49·7)
€18·001 - €100·000	1,941,353 (30·8)	1,932,463 (31·2)	1,887,750 (32·6)
>€100·000	49,313 (0·8)	48,970 (0·8)	47,912 (0·8)

**Income category (proxy), *n (%)***	5,691,196	5,637,000 (99·0)	5,335,598 (93·8)
Groups exempt from contribution (0%&)	540,299 (8·6)	539,007 (8·7)	522,617 (9·0)
Pensioners (no contribution limit)	388 (0)	379 (0)	180 (0)
Pensioners – income <€18,000 (up to €8/month)	756,277 (12·0)	755,652 (12·2)	584,671 (10·1)
Pensioners – income range €18,000 - €100,000 (up to €18/m)	471,572 (7·5)	471,182 (7·6)	435,852 (7·5)
Workers – income <€18,000 (40%)	2,403,566 (38·1)	2,360,529 (38·1)	2,292,468 (39·6)
Workers – income range €18,000 - €100,000 (50%)	1,438,413 (22·8)	1,430,232 (23·1)	1,422,045 (24·6)
Workers – income >€100,000 (60%)	39,192 (0·6)	38,868 (0·6)	38,456 (0·7)
Pensioners – income >€100,000 (up to €61/month)	10,121 (0·2)	10,102 (0·2)	9456 (0·2)
Others – income range €18,000 - €100,000 (excluded)	31,368 (0·5)	31,049 (0·5)	29,853 (0·5)
Missing	609,899 (9·7)	552,545 (8·9)	448,685 (7·8)
**Chronic conditions**			
**T2D, *n (%)***	783,870 (12·4)	741,178 (11·8)	633,370 (10·1)
**HBP, *n (%)***	1,355,894 (21·5)	1,312,804 (20·8)	1,115,290 (17·7)
**HF, *n (%)***	295,221 (4·7)	274,893 (4·4)	201,519 (3·2)
**Smoking status, *n (%)***			
Current smokers	NA	999,816 (15·9)	961,241 (15·3)
Former smokers	NA	969,029 (15·4)	1,006,596 (16·0)
Non-smokers	NA	2,674,546 (42·4)	2,668,483 (42·4)
Unknown status	NA	1,035,637 (16·4)	969,396 (15·4)

Abbreviations: HBP, high blood pressure; HF, heart failure; NA, not available; T2D, type 2 diabetes mellitus.

Table S5. Number of deaths among those diagnosed with some chronic conditions and average annual mortality rate (per 1,000 people diagnosed

	Deceased (crude mortality rate – annual average)		
**Diagnosis**	**All period** *n (%)* January 1, 2015-June 30, 2021	**Pre-pandemic** *n (%)* January 1, 2015- March 13, 2020	**Pandemic** *n (%)* March 14, 2020-June 30, 2021
**type 2 Diabetes**	**120,326 (2.37%)**	**90,163 (2.34%)**	**30,163 (3.72%)**
**High blood pressure**	**218,803 (2.48%)**	**165,077 (2.42%)**	**53,726 (3.72%)**
**Heart failure**	**89,234 (4.65%)**	**68,963 (4.82%)**	**20,271 (7.82%)**

Table S6. Comparison of health indicators before and during the pandemic in the population without HBP overall and stratified by sex.

Health indicators (mean, SD)	Pre-pandemic	Pandemic	Difference*	Pre-pandemic (males)	Pandemic (males)	Difference* (males)	Pre-pandemic (females)	Pandemic (females)	Difference* (females)
**Total cholesterol**<br>n=821,503<br>*Males=309,090*<br>*Females=512,413*	196.1 (41.7)	199.7 (41.7)	3.6 (1.8%)	190.9 (42)	192.8 (42)	1.9 (1%)	199.3 (41.1)	203.9 (41.1)	4.6 (2.3%)
**HDL-C**<br>n=496,741<br>*Males=194,771*<br>*Females=301,970*	57.7 (15.1)	59.2 (15.1)	1.5 (2.6%)	50.7 (12.6)	52 (12.6)	1.3 (2.5%)	62.1 (14.9)	63.8 (14.9)	1.7 (2.7%)
**LDL-C**<br>n=496,486<br>*Males=194,603*<br>*Females=301,883*	123.3 (35.8)	123.9 (35.8)	0.6 (0.5%)	119.4 (36.4)	118.7 (36.4)	-0.7 (-0.6%)	125.8 (35.2)	127.2 (35.2)	1.4 (1.1%)
**Fasting blood glucose**<br>n=911,551<br>*Males=344,723*<br>*Females=566,828*	94.5 (26.2)	97.5 (26.2)	3.0 (3.2%)	99.6 (31.3)	102.8 (31.3)	3.2 (3.2%)	91.3 (21.9)	94.2 (21.9)	2.9 (3.2%)
**Hba1c,**<br>n=148,351<br>*Males=73,906*<br>*Females=74,445*	6.5 (1.4)	6.6 (1.4)	0.1 (2.0%)	6.7 (1.4)	6.8 (1.4)	0.1 (2%)	6.3 (1.3)	6.4 (1.3)	0.1 (1.9%)
**BMI**<br>n=413,174<br>*Males=158,830 Females=254,344*	26.2 (5.7)	26.8 (5.7)	0.6 (2.5%)	26.2 (5.4)	26.7 (5.4)	0.5 (2%)	26.2 (5.9)	26.9 (5.9)	0.7 (2.8%)
**SBP**<br>n=721,059<br>*Males=294,187 Females=426,872*	119.7 (15.6)	122.9 (15.6)	3.2 (2.6%)	122.1 (16)	125.5 (16)	3.3 (2.7%)	118.1 (15.1)	121.1 (15.1)	3 (2.6%)
**DBP**<br>n=721,061<br>*Males=294,189*<br>*Females=426,872*	72.3 (10.6)	74.5 (10.6)	2.2 (3.1%)	72.8 (11.1)	75.2 (11.1)	2.4 (3.3%)	71.9 (10.1)	74 (10.1)	2.1 (2.9%)

**Triglycerides**<br>n=576,180<br>*Males=234,762*<br>*Females=341,418*	123 (83.3)	127.1 (83.3)	4.2 (3.4%)	139.3 (102.7)	141.6 (102.7)	2.2 (1.6%)	111.7 (64.4)	117.2 (64.4)	5.5 (4.9%)
**REGICOR index**<br>n=89,302<br>*Males=38,800*<br>*Females=50,502*	4.1 (2.9)	4.4 (2.9)	0.4 (9.5%)	5.2 (3.4)	5.8 (3.4)	0.6 (11.4%)	3.2 (2.1)	3.4 (2.1)	0.2 (7.1%)
**Barthel index**<br>n=17,750<br>*Males=7349*<br>*Females=10,401*	66.6 (31.9)	56.1 (31.9)	-10.5 (-15.8%)	70.6 (31.3)	60.6 (31.3)	-10 (-14.1%)	63.8 (32.1)	52.9 (32.1)	-10.9 (-17.1%)

Abbreviations: BMI, body mass index; DBP, diastolic blood pressure; Hba1c, glycosylated hemoglobin; HBP, high blood pressure; HDL-C, high-density lipoprotein cholesterol; LDL-C, high-density lipoprotein cholesterol; SBP, systolic blood pressure.
*Wilcoxon test of the difference of the medians p<0.001 for all comparisons.

Table S7. Comparison of health indicators in the population with HBP diagnosed before the pandemic overall and stratified by sex.

Health indicators (mean, SD)	Pre-pandemic	Pandemic	Difference*	Pre-pandemic (males)	Pandemic (males)	Difference** (males)	Pre-pandemic (females)	Pandemic (females)	Difference*** (females)
**Total cholesterol**, n=662,561 *Males=308,219* *Females=354,342*	194.7 (41.7)	193.4 (41.7)	-1.2 (-0.6%)	183.9 (40.8)	181.9 (40.8)	-2.1 (-1.1%)	204 (40.1)	203.5 (40.1)	-0.5 (-0.2%)
**HDL-C** n=533,305 *Males=248,556* *Females=284,749*	54.1 (14.1)	54.9 (14.1)	0.7 (1.4%)	49.2 (12.4)	49.9 (12.4)	0.7 (1.5%)	58.4 (14.1)	59.2 (14.1)	0.8 (1.3%)
**LDL-C** n=532,795 *Males=248,193* *Females=284,602*	115.2 (35.6)	112.7 (35.6)	-2.5 (-2.1%)	108.3 (34.9)	105.4 (34.9)	-2.9 (-2.7%)	121.2 (35.2)	119.1 (35.2)	-2 (-1.7%)
**Fasting blood glucose** n=694,938 *Males=324,760* *Females=370,178*	110.7 (36.1)	114 (36.1)	3.3 (3%)	114.1 (37.9)	117.2 (37.9)	3.1 (2.7%)	107.6 (34.1)	111.1 (34.1)	3.5 (3.2%)
**Hba1c** n=297,803 *Males=153,164* *Females=144,639*	6.7 (1.2)	6.8 (1.2)	0.1 (1.8%)	6.7 (1.3)	6.8 (1.3)	0.1 (1.8%)	6.7 (1.2)	6.8 (1.2)	0.1 (1.9%)
**BMI** n=429,992 *Males=207,879* *Females=222,113*	29.7 (5.2)	29.6 (5.2)	-0.1 (-0.4%)	29.5 (4.7)	29.4 (4.7)	-0.2 (-0.5%)	30 (5.7)	29.9 (5.7)	-0.1 (-0.3%)
**SBP** n=626,137 *Males=295,236* *Females=330,901*	133.6 (13.9)	134 (13.9)	0.3 (0.2%)	134.1 (13.6)	134.2 (13.6)	0.1 (0.1%)	133.3 (14.2)	133.8 (14.2)	0.5 (0.4%)
**DBP**, n=626,185 *Males=295,252* *Females=330,933*	76.6 (10.3)	76.6 (10.3)	0 (0%)	77.3 (10.5)	77.2 (10.5)	-0.1 (-0.1%)	76 (10.1)	76.1 (10.1)	0.1 (0.1%)**
**Triglycerides** n=597,674 *Males=281,335* *Females=316,339*	139 (82.9)	142.1 (82.9)	3 (2.2%)	143.4 (94.5)	144.3 (94.5)	0.9 (0.7%)	135.1 (70.8)	140 (70.8)	4.9 (3.6%)

**REGICOR index**<br>n=155,458<br>*Males=80,726*<br>*Females=74,732*	5.2 (3.4)	5.5 (3.4)	0.3 (5.6%)	6.3 (3.7)	6.8 (3.7)	0.5 (8.2%)	4.1 (2.4)	4.1 (2.4)	0.1 (1.5%)
**Barthel index**<br>n=75,660<br>*Males=24,675*<br>*Females=50,985*	71.5 (27.5)	60.3 (27.5)	-11.2 (-15.7%)	77.2 (26.2)	66.4 (26.2)	-10.9 (-14.1%)	68.7 (27.7)	57.4 (27.7)	-11.4 (-16.6%)

Abbreviations: BMI, body mass index; DBP, diastolic blood pressure; Hba1c, glycosylated hemoglobin; HBP, high blood pressure; HDL-C, high-density lipoprotein cholesterol; LDL-C, high-density lipoprotein cholesterol; SBP, systolic blood pressure.
*Wilcoxon test of the difference of the medians $p<0.001$ for all comparisons, except for DBP p, not significant.
**Wilcoxon test of the difference of the medians $p<0.001$ for all comparisons.
***Wilcoxon test of the difference of the medians $p<0.001$ for all comparisons, except for DBP, $p<0.01$.

Table S8. Comparison of health indicators in the population with HBP diagnosed during the pandemic overall and stratified by sex.

Health indicators (mean, SD)	Pre-pandemic	Pandemic	Difference*	Pre-pandemic (males)	Pandemic (males)	Difference** (males)	Pre-pandemic (females)	Pandemic (females)	Difference* (females)
**Total cholesterol**<br>n=24,552<br>*Males=11,116 Females=13,436*	207.5 (40.9)	209.2 (40.9)	1.7 (0.8%)	200.5 (41.8)	200.8 (41.8)	0.3 (0.1%)	213.3 (39.1)	216.2 (39.1)	2.9 (1.4%)
**HDL-C**<br>n=18,207<br>*Males=8,134 Females=10,073*	55.9 (14.6)	56.9 (14.6)	1 (1.8%)	49.8 (12.4)	50.8 (12.4)	1 (1.9%)	60.7 (14.5)	61.8 (14.5)	1 (1.7%)
**LDL-C**<br>n=18,200<br>*Males=8,127 Females=10,073*	127.9 (35.2)	127.1 (35.2)	-0.8 (-0.6%)	123.3 (35.7)	121.7 (35.7)	-1.6 (-1.3%)	131.7 (34.3)	131.5 (34.3)	-0.2 (-0.2%)
**Blood glucose**<br>n=25,524<br>*Males=11,632*<br>*Females=13,892*	102.2 (31.4)	106.4 (31.4)	4.2 (4.1%)	106.8 (36.1)	110.9 (36.1)	4.1 (3.9%)	98.4 (26.3)	102.6 (26.3)	4.2 (4.3%)
**Hba1c**<br>n=6785<br>*Males=3526*<br>*Females=3259*	6.6 (1.4)	6.7 (1.4)	0.2 (2.4%)	6.8 (1.5)	6.9 (1.5)	0.2 (2.2%)	6.4 (1.2)	6.5 (1.2)	0.2 (2.5%)
**BMI**<br>n=19,655<br>*Males=9619 Females=10,036*	29.1 (5.3)	29.7 (5.3)	0.6 (2.2%)	28.9 (4.8)	29.6 (4.8)	0.7 (2.3%)	29.2 (5.7)	29.9 (5.7)	0.6 (2.2%)
**SBP**<br>n=32,113<br>*Males=15,581 Females=16,532*	135.1 (14.3)	138 (14.3)	2.9 (2.1%)	136.4 (14)	139.3 (14)	2.9 (2.1%)	134 (14.5)	136.8 (14.5)	2.9 (2.1%)
**DBP**<br>n=32,116<br>*Males=15,583 Females=16,533*	81.3 (10.3)	83.1 (10.3)	1.8 (2.2%)	82.3 (10.4)	84.4 (10.4)	2.1 (2.6%)	80.4 (10)	81.8 (10)	1.5 (1.8%)

**Triglycerides**<br>n=20,841<br>*Males=9603*<br>*Females=11,238*	139.5 (99.3)	145.6 (99.3)	6 (4.3%)	154.8 (119.4)	157.7 (119.4)	2.9 (1.9%)	126.5 (75.6)	135.1 (75.6)	8.7 (6.9%)
**REGICOR index**<br>n=6232<br>*Males=2982 Females=3250*	4.6 (3.1)	5.5 (3.1)	0.9 (20.3%)	5.6 (3.6)	7 (3.6)	1.3 (23.6%)	3.5 (2.2)	4.1 (2.2)	0.5 (15.4%)
**Barthel index**<br>n=653<br>*Males=225 Females=428*	76.2 (26.5)	66.4 (26.5)	-9.8 (-12.8%)	80.6 (25.4)	70.5 (25.4)	-10.1 (-12.5%)	73.9 (26.8)	64.3 (26.8)	-9.6 (-13%)

Abbreviations: BMI, body mass index; DBP, diastolic blood pressure; Hba1c, glycosylated hemoglobin; HBP, high blood pressure; HDL-C, high-density lipoprotein cholesterol; LDL-C, high-density lipoprotein cholesterol; SBP, systolic blood pressure.

*Wilcoxon test of the difference of the medians $p<0.001$ for all comparisons.

**Wilcoxon test of the difference of the medians $p<0.001$ for all comparisons, except for LDL $p<0.05$.

Table S9. Comparison of health indicators before and during the pandemic in the population without HF overall and stratified by sex

Health indicators (mean, SD)	Pre-pandemic	Pandemic	Difference*	Pre-pandemic (males)	Pandemic (males)	Difference** (males)	Pre-pandemic (females)	Pandemic (females)	Difference** (females)
**Total cholesterol**<br>n=1,372,150<br>*Males=566,178*<br>*Females=805,972*	196.8 (41.5)	198.7 (41.5)	1.9 (1%)	189.4 (41.4)	189.7 (41.4)	0.3 (0.2%)	202 (40.7)	205 (40.7)	3 (1.5%)
**HDL-C**<br>n=940,114<br>*Males=401,413*<br>*Females=538,701*	56.1 (14.7)	57.3 (14.7)	1.2 (2.1%)	50 (12.5)	51.1 (12.5)	1 (2.1%)	60.7 (14.6)	62 (14.6)	1.3 (2.2%)
**LDL-C**<br>n=939,492<br>*Males=400,980*<br>*Females=538,512*	120.7 (35.7)	120 (35.7)	-0.7 (-0.5%)	115.1 (35.8)	113.5 (35.8)	-1.6 (-1.4%)	124.8 (35.1)	124.9 (35.1)	0.1 (0%)
**Fasting blood glucose**<br>n=1,487,099<br>*Males=614,779*<br>*Females=872,320*	100.6 (31.2)	103.8 (31.2)	3.2 (3.2%)	105.9 (35.1)	109.1 (35.1)	3.2 (3.1%)	96.8 (27.5)	100 (27.5)	3.1 (3.2%)
**Hba1c**,<br>n=387,814<br>*Males=197,641*<br>*Females=190,173*	6.6 (1.3)	6.7 (1.3)	0.1 (1.9%)	6.7 (1.3)	6.8 (1.3)	0.1 (1.9%)	6.5 (1.3)	6.6 (1.3)	0.1 (2%)
**BMI**<br>n=771,248<br>*Males=332,284*<br>*Females=438,964*	27.9 (5.7)	28.2 (5.7)	0.3 (1.2%)	27.9 (5.3)	28.2 (5.3)	0.2 (0.7%)	27.8 (6.1)	28.2 (6.1)	0.4 (1.5%)
**SBP**,<br>n=1,243,774<br>*Males=542,070*<br>*Females=701,704*	125.9 (16.4)	128 (16.4)	2.1 (1.6%)	128 (16.1)	130 (16.1)	2 (1.6%)	124.2 (16.5)	126.4 (16.5)	2.2 (1.7%)
**DBP**,<br>n=1,243,821<br>*Males=542,090*<br>*Females=701,731*	74.6 (10.7)	75.9 (10.7)	1.3 (1.8%)	75.4 (11.1)	76.8 (11.1)	1.3 (1.8%)	74 (10.3)	75.3 (10.3)	1.3 (1.8%)

**Triglycerides**<br>n=1,073,454<br>*Males=469,479*<br>*Females=603,975*	131.1 (84.6)	134.8 (84.6)	3.8 (2.9%)	142.8 (100.5)	144.4 (100.5)	1.7 (1.2%)	122 (68.5)	127.4 (68.5)	5.4 (4.4%)
**REGICOR index**<br>n=230,653<br>*Males=110,911*<br>*Females=119,742*	4.7 (3.2)	5.1 (3.2)	0.4 (7.4%)	5.9 (3.6)	6.4 (3.6)	0.6 (9.7%)	3.7 (2.3)	3.8 (2.3)	0.2 (4.2%)
**Barthel index**<br>n=62,939<br>*Males=21,587 Females=41,352*	69.4 (29.8)	58.5 (29.8)	-11 (-15.8%)	74.1 (29)	63.8 (29)	-10.3 (-13.9%)	67 (29.9)	55.7 (29.9)	-11.3 (-16.9%)

Abbreviations: BMI, body mass index; DBP, diastolic blood pressure; Hba1c, glycosylated hemoglobin; HDL-C, high-density lipoprotein cholesterol; HF, heart failure; LDL-C, high-density lipoprotein cholesterol; SBP, systolic blood pressure.
*Wilcoxon test of the difference of the medians $p<0.001$ for all comparisons, except for LDL $p<0.05$.
**Wilcoxon test of the difference of the medians $p<0.001$ for all comparisons.

Table S10. Comparison of health indicators in the population with HF diagnosed before the pandemic overall and stratified by sex.

Health indicators (mean, SD)	Pre-pandemic	Pandemic	Difference*	Pre-pandemic (males)	Pandemic (males)	Difference** (males)	Pre-pandemic (females)	Pandemic (females)	Difference* (females)
**Total cholesterol**<br>n=122,173<br>*Males=55,613 Females=66,560*	183.8 (42.1)	181.2 (42.1)	-2.6 (-1.4%)	171.5 (39.8)	168.4 (39.8)	-3.1 (-1.8%)	194 (41.3)	192 (41.3)	-2.1 (-1.1%)
**HDL-C**<br>n=96,919<br>*Males=44,760 Females=52,159*	53.1 (14.4)	53.5 (14.4)	0.4 (0.7%)	48.5 (12.8)	48.8 (12.8)	0.4 (0.7%)	57.2 (14.4)	57.5 (14.4)	0.4 (0.6%)
**LDL-C**<br>n=96,777<br>*Males=44,661*<br>*Females=52,116*	106.3 (35.6)	103 (35.6)	-3.4 (-3.2%)	98.6 (33.8)	94.7 (33.8)	-3.8 (-3.9%)	113 (35.7)	110 (35.7)	-3 (-2.6%)
**Fasting blood glucose**<br>n=129,690<br>*Males=59,242 Females=70,448*	110.6 (36.6)	113.6 (36.6)	3 (2.7%)	113.7 (37.7)	116.5 (37.7)	2.8 (2.4%)	107.9 (35.4)	111.1 (35.4)	3.2 (3%)
**Hba1c**<br>n=58,415<br>*Males=29,449*<br>*Females=28,966*	6.7 (1.2)	6.8 (1.2)	0.1 (1.6%)	6.7 (1.2)	6.8 (1.2)	0.1 (1.6%)	6.6 (1.2)	6.8 (1.2)	0.1 (1.6%)
**BMI**<br>n=81,372<br>*Males=39,050*<br>*Females=42,322*	29.5 (5.4)	29.3 (5.4)	-0.2 (-0.8%)	29.2 (4.9)	29 (4.9)	-0.3 (-0.9%)	29.7 (5.9)	29.5 (5.9)	-0.2 (-0.8%)
**SBP**<br>n=120,139<br>*Males=55,597*<br>*Females=64,542*	131 (15.2)	131.3 (15.2)	0.2 (0.2%)	130.8 (14.7)	130.9 (14.7)	0.2 (0.1%)*	131.3 (15.6)	131.5 (15.6)	0.3 (0.2%)
**DBP**<br>n=120,144<br>*Males=55,597*<br>*Females=64,547*	72.9 (10.4)	73.1 (10.4)	0.2 (0.3%)	73.3 (10.6)	73.4 (10.6)	0.2 (0.2%)	72.7 (10.3)	72.9 (10.3)	0.2 (0.3%)

**Triglycerides**<br>n=108,634<br>*Males=50,279*<br>*Females=58,355*	133.3 (75.3)	135.8 (75.3)	2.5 (1.9%)	133.5 (81.9)	134.5 (81.9)	1 (0.8%)	133.2 (69.2)	137 (69.2)	3.8 (2.8%)
**REGICOR index**<br>n=18,036<br>*Males=10,296*<br>*Females=7740*	5.4 (3.5)	5.6 (3.5)	0.2 (4.6%)	6.3 (3.7)	6.8 (3.7)	0.5 (7.8%)	4.1 (2.6)	4.1 (2.6)	-0.1 (-1.7%)
**Barthel index**<br>n=28,240<br>*Males=9671*<br>*Females=18,569*	72.9 (25.5)	62 (25.5)	-11 (-15%)	78.9 (24.3)	68 (24.3)	-10.9 (-13.8%)	69.8 (25.6)	58.8 (25.6)	-11 (-15.7%)

Abbreviations: BMI, body mass index; DBP, diastolic blood pressure; Hba1c, glycosylated hemoglobin; HDL-C, high-density lipoprotein cholesterol; HF, heart failure; LDL-C, high-density lipoprotein cholesterol; SBP, systolic blood pressure.
*Wilcoxon test of the difference of the medians $p<0.001$ for all comparisons.
**Wilcoxon test of the difference of the medians $p<0.001$ for all comparisons, except for SBP $p<0.05$.

Table S11. Comparison of health indicators in the population with HF diagnosed during the pandemic overall and stratified by sex.

Health indicators (mean, SD)	Pre-pandemic	Pandemic	Difference*	Pre-pandemic (males)	Pandemic (males)	Difference** (males)	Pre-pandemic (females)	Pandemic (females)	Difference*** (females)
**Total cholesterol**, n=14,293 *Males=6634* *Females=7659*	189.4 (42.4)	181.2 (42.4)	-8.2 (-4.3%)	178.7 (41.1)	168.4 (41.1)	-10.3 (-5.8%)	198.6 (41.4)	192.2 (41.4)	-6.4 (-3.2%)
**HDL-C** n=11,220 *Males=5288,* *Females=5932*	54.4 (14.7)	53.5 (14.7)	-0.9 (-1.6%)	49.7 (13.4)	48.9 (13.4)	-0.8 (-1.6%)	58.7 (14.6)	57.7 (14.6)	-0.9 (-1.6%)
**LDL-C**, n=11,212 *Males=5282* *Females=5930*	111 (35.7)	103.5 (35.7)	-7.5 (-6.8%)	104.3 (34.5)	95.5 (34.5)	-8.8 (-8.4%)	117 (35.7)	110.7 (35.7)	-6.4 (-5.4%)
**Fasting blood glucose** n=15,224 *Males=7094* *Females=8130*	111.4 (39.5)	114.8 (39.5)	3.3 (3%)	115.1 (40.6)	117.4 (40.6)	2.3 (2%)	108.2 (38.3)	112.4 (38.3)	4.2 (3.9%)
**Hba1c** n=6710 *Males=3506* *Females=3204*	6.7 (1.3)	6.8 (1.3)	0.1 (1.4%)	6.8 (1.3)	6.8 (1.3)	0.1 (0.9%)	6.7 (1.3)	6.8 (1.3)	0.1 (2%)
**BMI** n=10,201 *Males=4994* *Females=5207*	29.8 (5.6)	29.5 (5.6)	-0.3 (-1%)	29.5 (5.1)	29.1 (5.1)	-0.4 (-1.4%)	30.2 (6.1)	30 (6.1)	-0.2 (-0.7%)
**SBP** n=15,396 *Males=7337* *Females=8059*	133.6 (15.5)	131 (15.5)	-2.5 (-1.9%)	133.9 (15.5)	130.8 (15.5)	-3 (-2.3%)	133.3 (15.6)	131.2 (15.6)	-2.1 (-1.6%)
**DBP** n=15,397 *Males=7337* *Females=8060*	74.6 (10.8)	73.6 (10.8)	-1 (-1.3%)	75.1 (11.3)	73.8 (11.3)	-1.3 (-1.7%)	74.1 (10.3)	73.5 (10.3)	-0.7 (-0.9%)

**Triglycerides**<br>n=12,607<br>*Males=5942*<br>*Females=6665*	132.5 (79.2)	134.2 (79.2)	1.7 (1.3%)	134.8 (88.2)	132.7 (88.2)	-2.1 (-1.5%)	130.5 (70.2)	135.5 (70.2)	5 (3.8%)
**REGICOR index**<br>n=2303<br>*Males=1301*<br>*Females=1002*	5.6 (3.8)	5.7 (3.8)	0.1 (2%)	6.7 (4.2)	6.9 (4.2)	0.2 (3.5%)	4.1 (2.7)	4.1 (2.7)	0 (-1%)
**Barthel index**<br>n=2884<br>*Males=991*<br>*Females=1893*	74.3 (24.5)	59.6 (24.5)	-14.6 (-19.7%)	80.7 (22.3)	65.9 (22.3)	-14.9 (-18.4%)	70.9 (24.9)	56.3 (24.9)	-14.5 (-20.5%)

Abbreviations: BMI, body mass index; DBP, diastolic blood pressure; Hba1c, glycosylated hemoglobin; HDL-C, high-density lipoprotein cholesterol; HF, heart failure; LDL-C, high-density lipoprotein cholesterol; SBP, systolic blood pressure.
*Wilcoxon test of the difference of the medians $p<0.001$ for all comparisons, except for REGICOR $p<0.05$.
**Wilcoxon test of the difference of the medians $p<0.001$ for all comparisons, except for triglycerides *p*, not significant and REGICOR $p<0.01$.
**Wilcoxon test of the difference of the medians $p<0.001$ for all comparisons, except for REGICOR *p*, not significant.

Table S12. Comparison of health indicators before and during the pandemic in the population without T2D overall and stratified by sex.

Health indicators (mean, SD)	Pre-pandemic	Pandemic	Difference*	Pre-pandemic (males)	Pandemic (males)	Difference* (males)	Pre-pandemic (females)	Pandemic (females)	Difference* (females)
**Total cholesterol** n=1,073,039 *Males=406,077* *Females=666,962*	198.7 (41.2)	201.3 (41.2)	2.6 (1.3%)	192.2 (41.1)	193.3 (41.1)	1.1 (0.6%)	202.6 (40.8)	206.1 (40.8)	3.5 (1.7%)
**HDL-C** n=692,731 *Males=268,129* *Females=424,602*	58.2 (14.8)	59.4 (14.8)	1.2 (2.1%)	51.5 (12.6)	52.6 (12.6)	1.1 (2.1%)	62.4 (14.6)	63.7 (14.6)	1.3 (2.2%)
**LDL-C** n=692,457 *Males=267,970* *Females=424,487*	124.5 (35.2)	124.4 (35.2)	0.0% (0.0%)	120.2 (35.5)	119.2 (35.5)	-1.0 (-0.9%)	127.2 (34.8)	127.8 (34.8)	0.6 (0.5%)
**Fasting blood glucose** n=1,175,063 *Males=448,261,* *Females=726,802*	90.2 (14.3)	92.9 (14.3)	2.7 (3.0%)	92.3 (15.7)	95.1 (15.7)	2.8 (3.0%)	88.9 (13.3)	91.6 (13.3)	2.7 (3.0%)
**Hba1c** n=100,958 *Males=43,945 Females=57,013*	5.7 (0.8)	5.8 (0.8)	0.1 (1.2%)	5.7 (0.8)	5.8 (0.8)	0.1 (1.3%)	5.6 (0.7)	5.7 (0.7)	0.1 (1.1%)
**BMI** n=556,201 *Males=215,430* *Females=340,771*	26.9 (5.6)	27.4 (5.6)	0.5 (1.9%)	26.9 (5.3)	27.3 (5.3)	0.4 (1.6%)	26.8 (5.8)	27.4 (5.8)	0.5 (2.0%)
**SBP** n=982,344 *Males=400,165* *Females=582,179*	123.9 (16.6)	126.3 (16.6)	2.3 (1.9%)	125.8 (16.6)	128.2 (16.6)	2.4 (1.9%)	122.7 (16.5)	125.0 (16.5)	2.3 (1.9%)
**DBP** n=982,371 *Males=400,175* *Females=582,196*	73.9 (11.0)	75.5 (11.0)	1.6 (2.1%)	74.6 (11.6)	76.3 (11.6)	1.7 (2.3%)	73.4 (10.5)	74.9 (10.5)	1.5 (2.0%)

**Triglycerides**<br>n=794,496<br>*Males=317,956*<br>*Females=476,540*	120.5 (72.6)	124.4 (72.6)	3.9 (3.3%)	132.5 (88.8)	134.5 (88.8)	2.0 (1.5%)	112.5 (58)	117.7 (58)	5.2 (4.7%)
**REGICOR index**<br>n=134,610<br>*Males=56,160*<br>*Females=78,450*	3.7 (2.3)	3.9 (2.3)	0.2 (6.3%)	4.8 (2.8)	5.2 (2.8)	0.4 (9.3%)	2.9 (1.5)	2.9 (1.5)	0.1 (2.8%)
**Barthel index**<br>n=51,848<br>*Males=15,683 Females=36,165*	69 (29.4)	57.8 (29.4)	-11.2 (-16.2%)	73.9 (28.8)	63.2 (28.8)	-10.7 (-14.5%)	66.9 (29.3)	55.5 (29.3)	-11.4 (-17.1%)

Abbreviations: BMI, body mass index; DBP, diastolic blood pressure; Hba1c, glycosylated hemoglobin; HDL-C, high-density lipoprotein cholesterol; LDL-C, high-density lipoprotein cholesterol; SBP, systolic blood pressure; T2D, type 2 diabetes

*Wilcoxon test of the difference of the medians $p<0.001$ for all comparisons.

Table S13. Comparison of health indicators in the population with T2D diagnosed before the pandemic and stratified by sex.

Health indicators (mean, SD)	Pre-pandemic	Pandemic	Difference*	Pre-pandemic (males)	Pandemic (males)	Difference** (males)	Pre-pandemic (females)	Pandemic (females)	Difference** (females)
**Total cholesterol**<br>n=406,196<br>*Males=208,245*<br>*Females=197,951*	187.2 (41.6)	185.7 (41.6)	-1.4 (-0.8%)	178.2 (40.7)	175.9 (40.7)	-2.3 (-1.3%)	196.6 (40.4)	196.1 (40.4)	-0.6 (-0.3%)
**HDL-C**<br>n=333,081<br>*Males=172,657*<br>*Females=160,424*	51.2 (13.3)	52 (13.3)	0.8 (1.6%)	47.5 (12)	48.2 (12)	0.8 (1.6%)	55.2 (13.4)	56.1 (13.4)	0.9 (1.6%)
**LDL-C**<br>n=332,598<br>*Males=172,289*<br>*Females=160,309*	108.0 (35.0)	105.2 (35)	-2.8 (-2.6%)	102.3 (33.9)	99.1 (33.9)	-3.2 (-3.1%)	114.2 (35)	111.8 (35)	-2.4 (-2.1%)
**Fasting blood glucose**<br>n=425,621<br>*Males=218,197*<br>*Females=207,424*	132.1 (44.6)	135.7 (44.6)	3.6 (2.7%)	135.9 (45.7)	139 (45.7)	3.1 (2.3%)	128.1 (43.1)	132.2 (43.1)	4.0 (3.1%)
**Hba1c**<br>n=342,009<br>*Males=181,829*<br>*Females=160,180*	6.9 (1.3)	7.0 (1.3)	0.1 (1.9%)	7.0 (1.3)	7.1 (1.3)	0.1 (1.8%)	6.9 (1.3)	7 (1.3)	0.1 (2%)
**BMI**<br>n=284,800<br>*Males=150,686*<br>*Females=134,114*	30.1 (5.3)	29.9 (5.3)	-0.2 (-0.7%)	29.6 (4.7)	29.4 (4.7)	-0.3 (-0.9%)	30.7 (5.8)	30.5 (5.8)	-0.2 (-0.6%)
**SBP**<br>n=369,233<br>*Males=191,633*<br>*Females=177,600*	132.6 (13.9)	133.4 (13.9)	0.7 (0.6%)	133.3 (13.3)	133.9 (13.3)	0.6 (0.4%)	131.9 (14.4)	132.8 (14.4)	0.9 (0.7%)
**DBP**<br>n=369,258<br>*Males=191,643*<br>*Females=177,615*	75.7 (9.8)	75.9 (9.8)	0.2 (0.3%)	76.2 (9.9)	76.3 (9.9)	0.1 (0.2%)	75.1 (9.7)	75.5 (9.7)	0.3 (0.4%)

**Triglycerides**<br>n=374,421<br>*Males=195,122*<br>*Females=179,299*	152.5 (98.6)	155.2 (98.6)	2.7 (1.8%)	155.2 (109.7)	155.9 (109.7)	0.6 (0.4%)	149.6 (84.7)	154.5 (84.7)	4.9 (3.3%)
**REGICOR index**<br>n=108,746<br>*Males=62,460*<br>*Females=46,286*	6.2 (3.7)	6.6 (3.7)	0.4 (6.8%)	7.0 (4.0)	7.6 (4.0)	0.6 (8.6%)	5.1 (2.8)	5.3 (2.8)	0.2 (3.4%)
**Barthel index**<br>n=41,346<br>*Males=16,261 Females=25,085*	72.5 (27.2)	61.6 (27.2)	-10.9 (-15.1%)	77.4 (26.3)	66.8 (26.3)	-10.6 (-13.7%)	69.3 (27.4)	58.2 (27.4)	-11.1 (-16.1%)

Abbreviations: BMI, body mass index; DBP, diastolic blood pressure; Hba1c, glycosylated hemoglobin; HDL-C, high-density lipoprotein cholesterol; LDL-C, high-density lipoprotein cholesterol; SBP, systolic blood pressure; T2D, type 2 diabetes.

*Wilcoxon test of the difference of the medians $p<0.001$ for all comparisons, except for the Barthel index p, not significant.

**Wilcoxon test of the difference of the medians $p<0.001$ for all comparisons.

Table S14. Comparison of health indicators in the population with T2D diagnosed during the pandemic and stratified by sex.

Health indicators (mean, SD)	Pre-pandemic	Pandemic	Difference*	Pre-pandemic (males)	Pandemic (males)	Difference** (males)	Pre-pandemic (females)	Pandemic (females)	Difference* (females)
**Total cholesterol** n=29,381 *Males=14,103 Females=15,278*	204.0 (42.1)	203.7 (42.1)	-0.2 (-0.1%)	197.6 (42.9)	195.7 (42.9)	-1.9 (-0.9%)	209.8 (40.5)	211.1 (40.5)	1.3 (0.6%)
**HDL-C** n=22,441 *Males=10,675 Females=11,766*	52.5 (13.4)	53.7 (13.4)	1.1 (2.1%)	47.7 (11.5)	48.9 (11.5)	1.2 (2.5%)	56.9 (13.5)	58.0 (13.5)	1.0 (1.8%)
**LDL-C** n=22,426 *Males=10,664 Females=11,762*	124.7 (35.6)	121.4 (35.6)	-3.3 (-2.7%)	120.0 (35.8)	115.6 (35.8)	-4.3 (-3.6%)	129.1 (34.9)	126.6 (34.9)	-2.5 (-1.9%)
**Fasting Blood glucose** n=31,329 *Males=14,657 Females=16,672*	107.6 (23.7)	122.4 (23.7)	14.8 (13.7%)	110.3 (25.7)	127.3 (25.7)	17 (15.4%)	105.3 (21.6)	118.1 (21.6)	12.8 (12.1%)
**Hba1c** n=9972 *Males=4822 Females=5150*	6.1 (0.8)	6.5 (0.8)	0.5 (7.4%)	6.1 (0.9)	6.6 (0.9)	0.5 (8.9%)	6.0 (0.8)	6.4 (0.8)	0.4 (6%)
**BMI** n=21,820 *Males=10,212 Females=11,608*	30.6 (5.7)	31.2 (5.7)	0.5 (1.8%)	30.6 (5.2)	30.8 (5.2)	0.2 (0.8%)	30.7 (6.1)	31.5 (6.1)	0.8 (2.6%)
**SBP** n=27,732 *Males=13,206, Females=14,526*	131.1 (14.9)	131.1 (14.9)	0.0 (0.0%)	133.4 (14)	133.0 (14.0)	-0.3 (-0.2%)	129.0 (15.4)	129.2 (15.4)	0.3 (0.2%)**
**DBP** n=27,733 *Males=13,206, Females=14,527*	78.6 (10.1)	78.7 (10.1)	0.1 (0.2%)	80.2 (10.1)	80.0 (10.1)	-0.1 (-0.2%)	77.1 (9.9)	77.5 (9.9)	0.4 (0.5%)

**Triglycerides**<br>n=25,778<br>*Males=12,622 Females=13,156*	155.6 (104.4)	163 (104.4)	7.4 (4.8%)	168.6 (124.1)	173.1 (124.1)	4.5 (2.7%)	143.2 (79.1)	153.3 (79.1)	10.2 (7.1%)
**REGICOR index**<br>n=7636<br>*Males=3888, Females=3748*	4.3 (2.6)	5.5 (2.6)	1.2 (27.8%)	5.2 (3.0)	6.7 (3.0)	1.5 (28.8%)	3.4 (1.8)	4.3 (1.8)	0.9 (26%)
**Barthel index**<br>n=869<br>*Males=305*<br>*Females=564*	78.0 (25.0)	67.4 (25.0)	-10.6 (-13.6%)	82.4 (23.6)	72.5 (23.6)	-10 (-12.1%)	75.7 (25.4)	64.7 (25.4)	-11.0 (-14.5%)

Abbreviations: BMI, body mass index; DBP, diastolic blood pressure; Hba1c, glycosylated hemoglobin; HDL-C, high-density lipoprotein cholesterol; LDL-C, high-density lipoprotein cholesterol; SBP, systolic blood pressure; T2D, type 2 diabetes.

*Wilcoxon test of the difference of the medians $p<0.001$ for all comparisons, except for SBP p, not significant, DBP $p<0.01$, and the Barthel index p, not significant.

**Wilcoxon test of the difference of the medians $p<0.001$ for all comparisons, except for cholesterol, SBP, and DBP p, not significant for the three comparisons.

Figure S1. Flow chart of the study cohort.

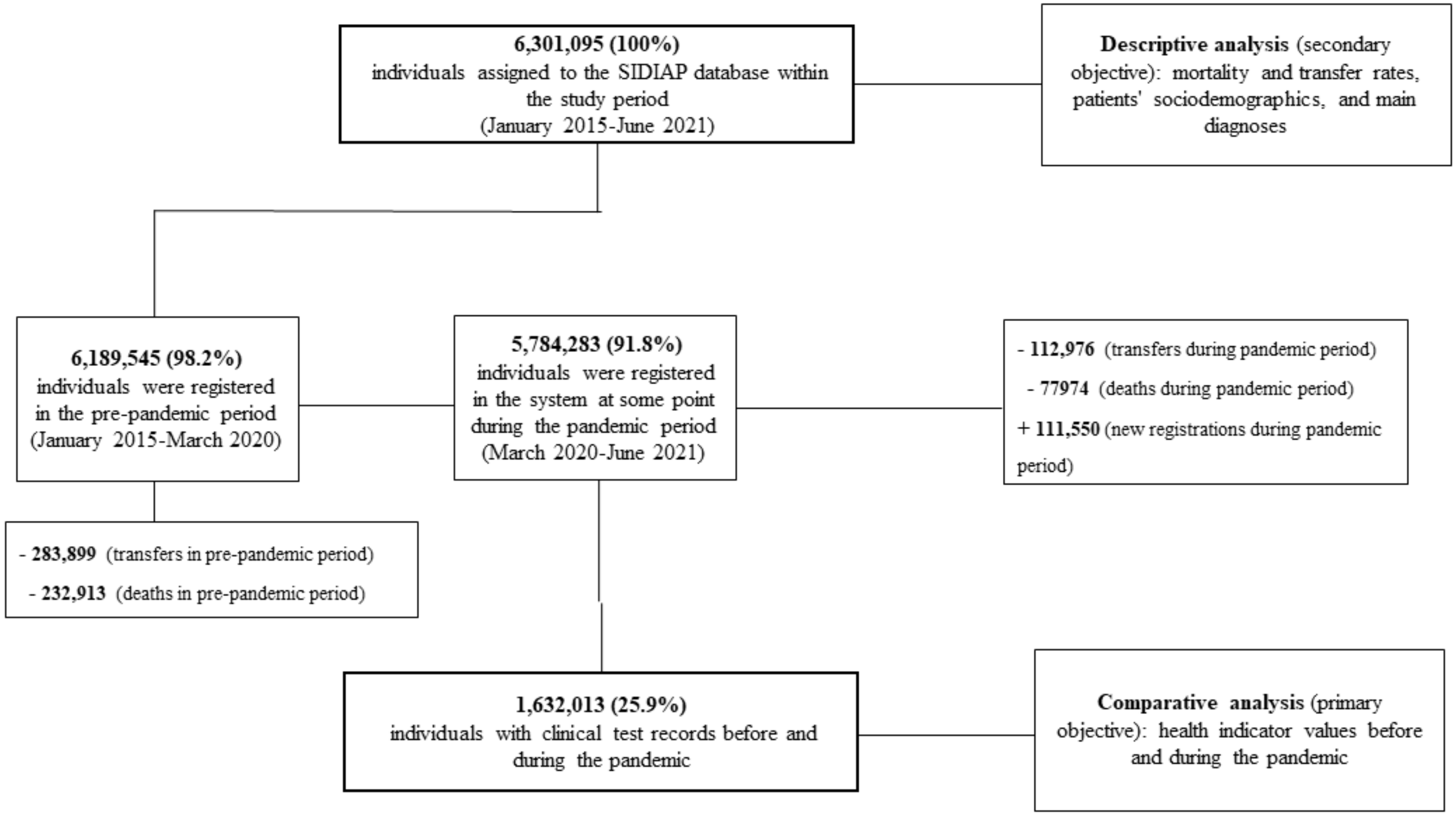

Figure S2. Representation of deaths from January 2018 to June 2021 (before and during the pandemic) for the total population (A); the population stratified by age groups (B); the population stratified by pharmaceutical co-payment (C); and the population stratified by MEDEA index quartiles (D). In (A), the line represents the crude death rate on the left axis, the bars represent death counts on the right axis for the total population, and the lines in (B-D) represent death rates.

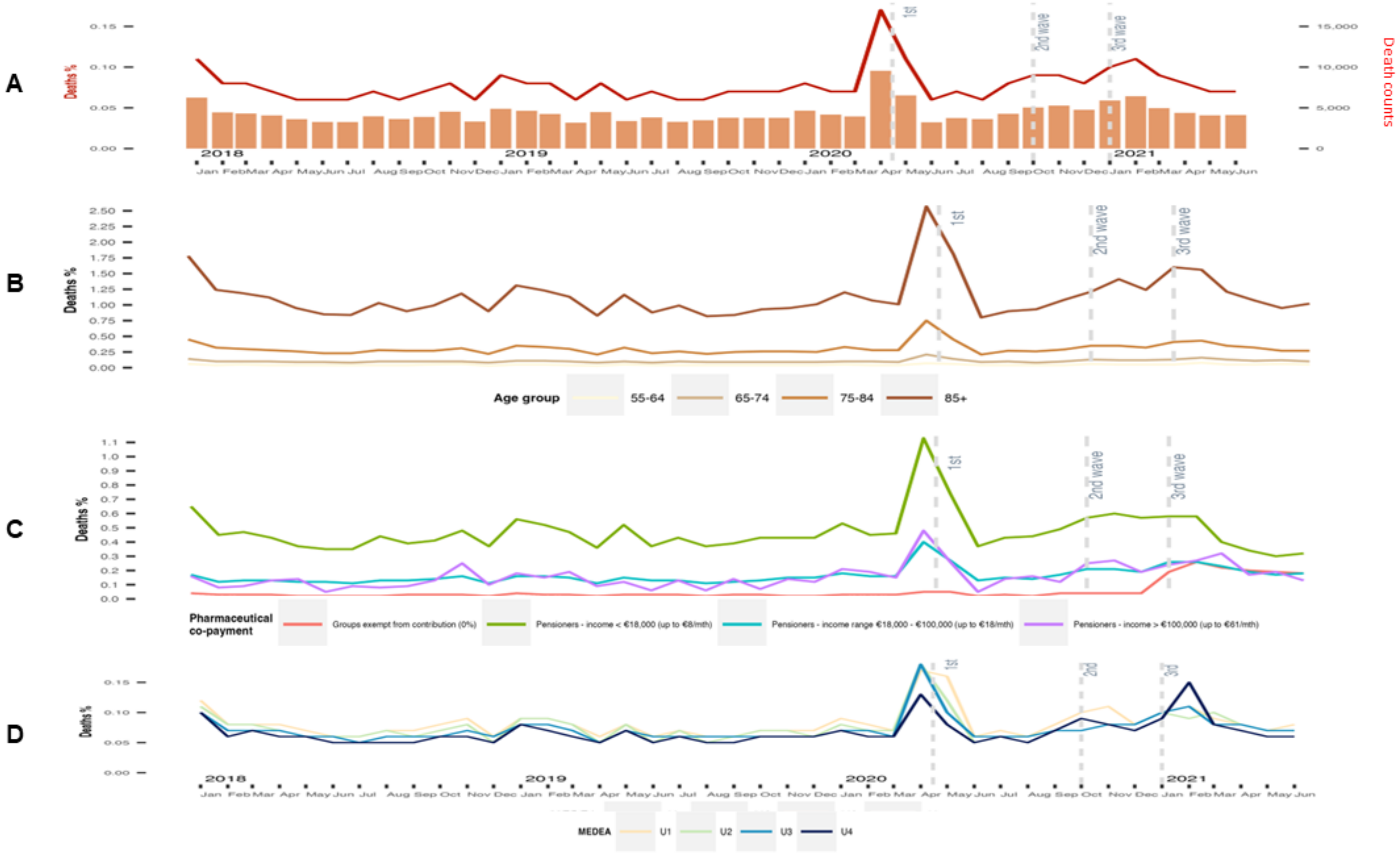

Figure S3. Representation of transfers from January 2018 to June 2021 (before and during the pandemic). For the total population (A) and the population stratified by age groups (B). In (A), the line represents the crude transfer rate on the left axis and the bars represent transfer counts on the right axis for the total population; the lines in (B) represent transfer rates.

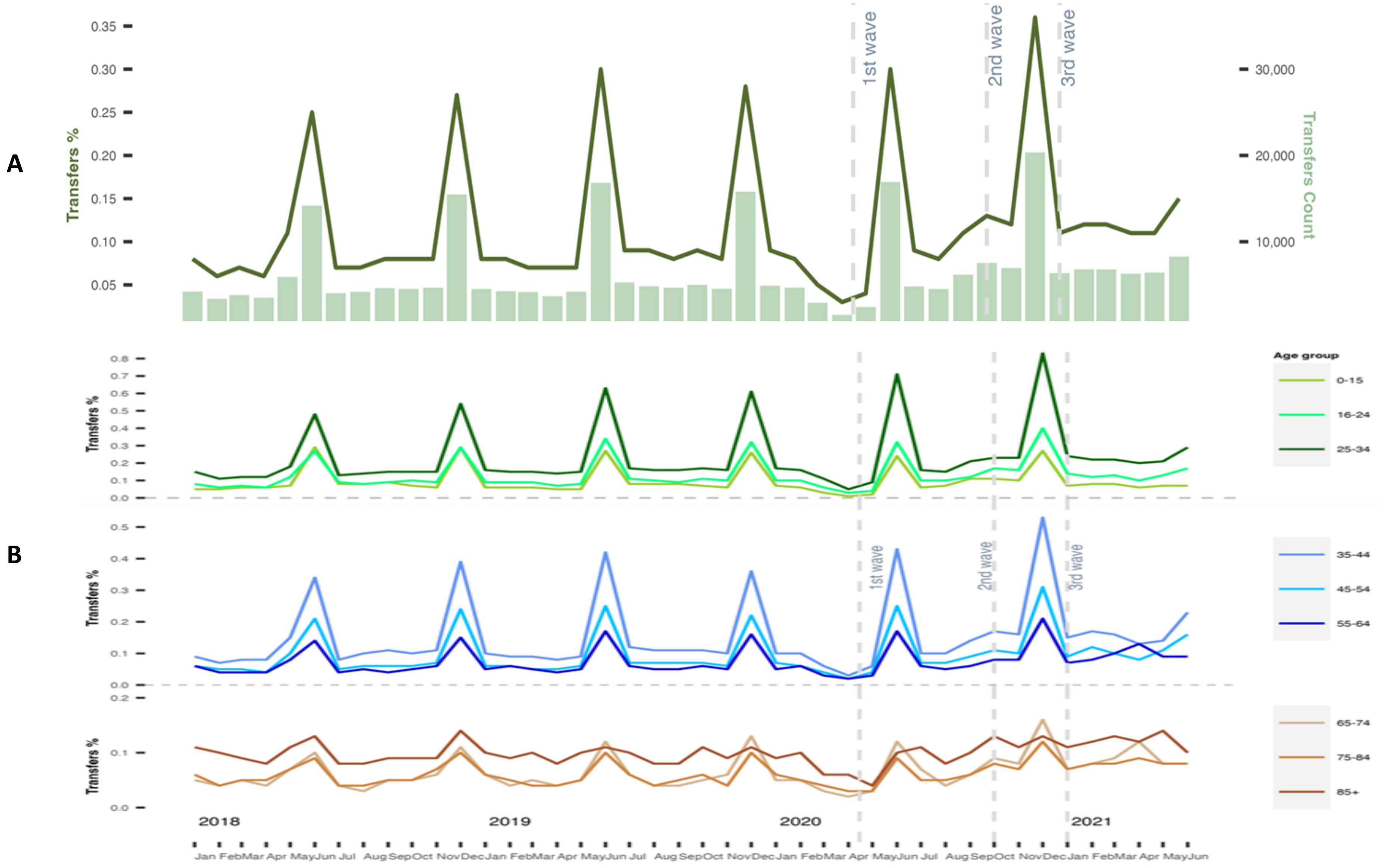

References

1 Benchimol EI, Smeeth L, Guttmann A, *et al.* The REporting of studies Conducted using Observational Routinely-collected health Data (RECORD) statement. *PLoS Med* 2015; **12**. DOI:10.1371/JOURNAL.PMED.1001885.

2 Recalde M, Rodríguez C, Burn E, *et al.* Data Resource Profile: The Information System for Research in Primary Care (SIDIAP). *Int J Epidemiol* 2022; **51**: e324–36.

3 Gresham GE, Phillips TF, Labi ML. ADL status in stroke: relative merits of three standard indexes. *Arch Phys Med Rehabil* 1980; **61**: 355–8.

4 Wylie CM. Measuring End Results of Rehabilitation of Patients with Stroke.

5 Marrugat J, Subirana I, Ramos R, *et al.* Derivation and validation of a set of 10-year cardiovascular risk predictive functions in Spain: the FRESCO Study. *Prev Med* 2014; **61**: 66–74.

6 Marrugat J, Vila J, Baena-Díez JM, *et al.* [Relative validity of the 10-year cardiovascular risk estimate in a population cohort of the REGICOR study]. *Rev Esp Cardiol* 2011; **64**: 385–94.

7 INE. Instituto Nacional de Estadística. Household Income Distribution Atlas. https://www.ine.es/dynt3/inebase/en/index.htm?padre=5608&capsel=5650.

8 INEbase / Demography and population /Municipal Register /Continuous Register Statistics / Results/ Final data. https://www.ine.es/dyngs/INEbase/en/operacion.htm?c=Estadistica_C&cid=1254736177012&menu=resultados&secc=1254736195461&idp=1254734710990#!tabs-1254736195557 (accessed June 21, 2023).

9 Catalan Health Service. Ministry of Health of the Government of Catalonia. The Catalan Information Systems Master Plan Building a digital health strategy for Catalonia together. 2017 https://catsalut.gencat.cat/web/.content/minisite/catsalut/actualitat/2021/documents/2021-05-14-The-Catalan-Information-System-Master-Plan.pdf (accessed June 23, 2023).
